# Natural flavonoid Orientin restricts 5-Fluorouracil induced cancer stem cells mediated angiogenesis by regulating HIF1α and VEGFA in colorectal cancer

**DOI:** 10.1186/s10020-024-01032-1

**Published:** 2025-03-05

**Authors:** Rituparna Ghosh, Arijit Bhowmik, Souradeep Biswas, Priya Samanta, Rupali Sarkar, Shampa Pakhira, Mrinmoyee Mondal, Subhadip Hajra, Prosenjit Saha

**Affiliations:** Department of Cancer Chemoprevention, Chittaranjan National Cancer Institute (CNCI), 37, Shyama Prasad Mukherjee Rd, Bakul Bagan, Bhowanipore, Kolkata, West Bengal 700026 India

**Keywords:** Angiogenesis, Cancer stem cell, Colorectal cancer, Orientin, 5-Fluorouracil, HIF1α, VEGFA, Natural flavonoid

## Abstract

**Background:**

Cancer stem cells are a small subpopulation of cells which are responsible for tumor metastasis, angiogenesis, drug resistance etc. 5-Fluorouracil (5FU), a common therapeutic drug used in colorectal cancer treatment is reported to enrich CSCs, tumor recurrence and induces severe organ toxicities resulting in poor clinical outcome in patients. Therefore, we introduced a natural flavonoid Orientin in combination with 5FU to mitigate the CSC mediated angiogenesis and induced toxicities.

**Methods:**

Tumorosphere generation, flow cytometry, immunofluorescence assay, and western blotting were performed by using 5FU and Orientin individually and both treated colorectal cells and CSCs*. *In silico study was carried out to check the interaction between HIF1α and Orientin. In ovo chorioallantoic membrane (CAM) assay and tube formation assay using HUVECs were performed to monitor CSC mediated angiogenesis. In vivo CT26 syngeneic mice model was used to validate in silico and ex vivo results.

**Results:**

We found that 5FU treatment significantly increased the CD44^+^/CD133^+^ CSC population. In contrast, this CSC population in CSC enriched spheres (CES) derived from HCT116 cells were decreased by combination of Orientin and 5FU. Decrease of CSC’s stemness properties was also noted, as evidenced by the downregulation of NANOG, SOX2 and OCT4. This new therapeutic strategy also inhibited CSC mediated angiogenesis by downregulating 5FU induced ROS, NO and LPO in those tumorospheres. Combination of Orientin and 5FU significantly reduced CSC mediated angiogenesis in HUVEC and CAM. Additionally, in silico study predicted that Orientin can bind to the PAS domain of HIF1α, a crucial factor for promoting angiogenesis. Expression of HIF1α and VEGFA were also decreased when the CESs were exposed to the combinatorial treatment. Additionally, we found that treatment with 5FU alone resulted reduction in tumor volume but it enriched CSCs and produced nephrotoxicity and hepatotoxicity in vivo. Combined treatment also considerably reduced the CD44^+^/CD133^+^ CSC population and hindered angiogenesis in a therapeutic in vivo model in BALB/c mice.

**Conclusions:**

This novel treatment strategy of "Orientin with 5FU" is likely to improve the efficiency of conventional chemotherapy and may suppress disease recurrence in colorectal cancer by limiting CSC mediated angiogenesis.

**Supplementary Information:**

The online version contains supplementary material available at 10.1186/s10020-024-01032-1.

## Background

Colorectal cancer (CRC) is the third most common malignancy worldwide (Colorectal cancer. 2024). According to Globocan 2022, CRC comprises of 9.6% of the total diagnosed cancer cases and has a high mortality rate (9.3% among all the cancers) due to rapid development of drug resistance and cancer recurrence (Bray et al. 2024). Besides, in India colorectal cancer holds the 4th rank in male (6.3% among all the cancers) and 5th rank (3.7% among all the cancers) in female considering the number of new cases in 2022. Considering the mortality rate, CRC cancer possess the 7th position and 4.5% among all the cancers (Today 2024). Mounting evidences suggest that, CSCs are considered as the tumor initiating cells and play an important role in drug resistance and recurrence of CRC, throwing major challenges in treatment of CRC (Samanta et al. 2023; Krishnapriya et al. 2019). CSCs are a group of tumor cells characterized by self-renewal, infinite proliferation, potential of multi-directional differentiation. These subpopulation of cells can promote angiogenesis by secreting proangiogenic factors such as Vascular Endothelial Growth Factor (VEGF) (Garza Treviño et al. 2019). Previous study suggested that tumor microenvironment enriched with CSCs express high level of VEGF which recruits endothelial progenitor cells to promote angiogenesis (Folkins et al. 2009). According to Bao et al. tumor in xenograft mice developed from CD133^+^ stem-cell-like-glioma cells (SCLGC) have elevated VEGF levels than the tumor developed from CD133^−^ SCLGC (Bao et al. 2006). Angiogenesis, one of the crucial hallmarks of cancer, not only provides adequate amount of nutrition to the malignant cells within the tumor niche but also helps them to migrate into distal organs (Cao et al. 2020). Thus, angiogenesis induced by CSCs plays a crucial role in inducing metastasis along with decreasing the overall survival rate of colorectal cancer patients.

The most important chemotherapeutic agent currently used in CRC treatment is the 5FU, a synthetic fluorinated pyrimidine analog. It has several disadvantages including systemic toxicity and development of drug resistance leading to tumor recurrence. (Alzahrani et al. 2023). Resistance of cells to cytotoxic agents like 5FU is a very complex phenomenon which occurs due to increased expression of multiple drug resistant associated proteins. These proteins induce efflux of anticancer agents out of the cells or by epigenetic modifications, upregulation and induction of CSC marker expressions, release of cytokines and various growth factors (Blondy et al. 2020). 5FU induces activation and enrichment of CSCs in the residual tumors, contributes to recurrence after treatment, which may be the key factor for relapse. Todaro et al. have demonstrated that 5FU failed to induce apoptosis in CD133^+^ cancer stem cells purified from the human colorectal cancer specimen, whereas, CD133^−^ malignant cells are sensitive to 5FU treatment (Todaro et al. 2007). However, more confirmatory evidences regarding this are required (Sethy and Kundu 2021). Another major drawback of 5FU is that it has the ability to stimulate angiogenesis, which in turn limits its clinical outcome (Albertsson et al. 2006). CSCs within the tumor niche are enriched by HIF1α which is also a key regulator of tumor angiogenesis (Ghosh et al. 2022). Transcriptional/translational regulation and stabilization of HIF1α is induced by generation of reactive oxygen species (ROS) (Movafagh et al. 2015; Pialoux et al. 2009). On the other hand, 5FU induces ROS production (Hwang et al. 2007). HIF1α is a crucial transcription factors which can upregulate various proteins responsible for CSC maintenance like OCT4, SOX2, NANOG (Ghosh et al. 2022). This transcription factor also promotes tumor angiogenesis by upregulating various angiogenic factors like VEGFA by binding at the HRE sequence present at their promoter region of genes (Ghosh et al. 2022). Therefore, it will be fascinating to check the role of HIF1α in 5FU induced CSC mediated angiogenesis in CRC. Besides, in this present study we search for an agents that can reduce the 5FU induced toxicity and can conquer the CSCs induced angiogenesis in a cost-effective way.

Orientin, a natural flavonoid is mainly found in Indian medicinal plants like Holy basil (*Ocimum sanctum*), which is reported to have free-radical scavenging activities, antiaging, antiviral, antibacterial, anti-inflammatory, vasodilatation and cardioprotective, radiation protective, neuroprotective, antidepressant-like, anti-adipogenesis, and anti-nociceptive effects (Lam et al. 2016; Devi et al. 1999; An et al. 2015) It would be of interest to explore the role of Orientin alone or in combination with 5FU if any on CSC-mediated tumor angiogenesis by targeting 5FU resistant CSCs.

## Methods

### Cell lines and cell culture

Human colorectal cancer cell line, HCT116 and mouse colorectal cancer cell line CT26 were cultured in DMEM medium (Gibco, USA) containing 10% heat inactivated FBS (Gibco, USA) and 1% antibiotic–antimycotic mixture (Gibco, USA) in a humidified incubator (5% CO_2_ at 37 °C). Human umbilical vein endothelial cells (HUVEC) were maintained in endothelial cell medium (ECM, ScienCell, USA) supplemented with 5% FBS and antibiotic–antimycotic mixture (Gibco, USA) (Zhang et al. 2017).

### Generation of tumorospheres and collection of conditioned media

HCT116 CRC cells were grown in ultra-low attachment plate in 3dGRO™ Spheroid Medium (Sigma-Aldrich, USA) for 7 days. Spheroids enriched with CSCs were considered as CSC enriched spheres (CESs) or commonly referred as tumorospheres. After 7 days, tumorospheres were subjected to different treatments for 24 h, as per experimental protocol. Media of tumorospheres of all the treatment groups were collected, centrifuged to remove the cell debris and suspended spheres, supernatants were collected and vacuum concentrated. These supernatants were referred as conditioned medium (CM) and were stored at − 20 °C until use.

### Chemicals

Orientin, (purity ≥ 97%, HPLC grade) and 5FU (purity ≥ 99%, HPLC grade) both were obtained from Sigma-Aldrich Chemicals Private Limited, USA and stored at 4 °C until use.

### Cell viability assay

HCT116 cells (1 × 10^3^ cells/well) were seeded in 96-well plates and cell viability was determined using MTT (Sigma-Aldrich, USA). HCT116 cells were treated with various concentrations of Orientin and 5FU individually and also in combination. MTT (0.5 mg/ml) was added to the cells after 48 h of treatment and incubated for 3 h in dark at 37 °C. The generated formazan complexes were dissolved in DMSO and the absorbance were measured at 550 nm using a spectrophotometer (Infinite® 200 PRO, TECAN, Switzerland) (Bhowmik et al. 2013).

### Atomic force microscopy (AFM) analysis

The cancer cells treated with Orientin, 5FU and both in combination were imaged with a reflex-coated gold cantilever in contact mode by AFM. The material properties and dimensions of the probe used in contact mode were as follows: resonance frequency of 13 kHz (± 4 kHz), force constant of 0.2 N/m (± 0.14 N/m), cantilever length of 450 µm (± 10 µm), cantilever width of 38 µm (± 5 µm), cantilever thickness of 2 µm (± 1 µm), tip radius of 5 nm (± 1 nm), and tip height of 17 µm (± 2 µm). The scan area depends on the size of the cancer cell and ranged from 20 × 10–30 × 20 µm^2^. Image processing and data analysis were performed using Picoview 1.10 software.

### Cytochrome c release assay

HCT116 cells were treated with Orientin, 5FU and their combination for 12 h and Cytochrome c release assay was performed as described previously (Bhowmik et al. 2013). In brief, cells were incubated in DMEM culture medium containing 100 nM Mitotracker Red (Thermo Fisher Scientific, USA) at 37 °C for 30 min prior to harvesting followed by fixation with 4% (w/v) paraformaldehyde and subsequently permeabilized with 0.2% Triton X-100. After blocking in 3% BSA (Himedia, India), cells were incubated overnight with primary antibody against Cytochrome c (Santa Cruz Biotechnology, USA) at 4 °C followed by incubation with Alexa Fluor 488-conjugated secondary antibody (Thermo Fisher Scientific, USA) and images were captured by a fluorescence microscope (Olympus BX53, Japan).

### Flow cytometry analysis using Annexin V/PI staining

Percentage of apoptotic cells were detected by Annexin V and propidium Iodide (PI) staining by flow cytometry analysis according to the protocols provided by manufacturer of the kit (Invitrogen, USA). In brief, cells were washed with PBS, suspended in binding buffer followed by incubation with Annexin V and PI. Then, the cells were analysed by using a flow cytometer (BD LSRFortessaTM X-20).

### Transfection of HIF1α

HCT116 cells were transiently transfected with HA-HIF1α-pcDNA3 plasmid (Addgene, USA, Plasmid #18949) by using lipofectamineTM 2000 (Thermo Fisher Scientific, USA) according to the manufacturer’s instructions. After 24 h of transfection, those cells were subjected to various experiments.

### Flow cytometry analysis of HIF1α positive cells in CSCs enriched tumorospheres

Single cell suspensions were prepared from tumorospheres, followed by cells were blocked with BSA and permeabilized before incubation with human anti-CD44-FITC (Biolegend, USA), human anti-CD133-PE (Biolegend, USA) and anti-HIF1α-Alexa fluor 647 (Biolegend, USA) for 2 h. Percentages of CD44^+^/CD133^+^ cell population were determined within tumorospheres and HIF1α positive cells were determined within the CD44^+^/CD133^+^ cell population using a flow cytometer (BD LSRFortessa™ X-20) and analyzed by BD FACSDiva™ software.

### Magnetic‐activated cell sorting (MACS)

CD44^+^/CD133^+^ cells were isolated using MACS LS column (Miltenyi Biotec, USA) after leveled with CD44 and CD133 antibody conjugated with microbeads (Miltenyi Biotec, USA) by following manufacture’s protocol and subjected to further analysis. Cells expressing both CD44 and CD133 were isolated using magnetic cell sorter (MACS) (Guerriero et al. 2017).

### Western blot analysis

Protein samples were extracted from treated and untreated cells and subjected to western blot analysis by standard protocol (Bhowmik et al. 2013). Cells were incubated with Brefeldin A (10 µg/ml) for 4 h before harvesting to determine VEGFA expression. Briefly, cells were lysed with RIPA buffer [50 mmol/L Tris–HCl (pH 7.8), 150 mmol/L NaCl, 1% Triton X-100, 0.1% SDS, 0.5% sodium deoxycholate, and 1 mmol/L EDTA] containing complete protease inhibitor cocktail. Equal amount of protein was electrophoresed on SDS-PAGE and electro transferred onto polyvinylidene difluoride (PVDF) membranes using Bio-Rad mini gel apparatus. The membranes were probed with OCT4 (Thermo Fisher Scientific, USA), SOX2 (AbCam, USA), NANOG (Thermo Fisher Scientific, USA), HIF1α (Thermo Fisher Scientific, USA) and VEGFA (Thermo Fisher Scientific, USA) and NF-ĸB (Santa Cruz Biotechnology, USA) antibodies. β-Actin (Santa Cruz Biotechnology, USA) and α-TUBULIN (Santa Cruz Biotechnology, USA) antibodies were used as loading control. Then, the blots were probed with HRP tagged secondary antibodies. Signals were detected with the ECL system (Promega, USA) and images were captured in iBright™ CL1500 Imaging System.

### ELISA

Concentration of the secreted VEGFA was measured using the ELISA kit according to the manufacturer’s instruction (Krishgen BioSystems, India) in CM of CESs as well as in the media of transfected and non-transfected HCT116.

### Real time PCR assay

Total RNA was extracted from sorted CD44^+^/CD133^+^ cell population using TRIZOL (Thermo Fisher Scientific, USA) as described in manufacturer’s protocol. cDNAs were synthesized from RNA templates by using cDNA isolation kit (Bio-Rad, USA) and target genes like OCT4, SOX2 and NANOG were amplified by using real time PCR kit (Bio-Rad, USA) following manufacturer’s instructions by Roche LightCycler® 96 System (Switzerland). Glyceraldehyde-3-phosphate dehydrogenase (GAPDH) gene expression was used to normalize the expression of the target genes. Sequences of the primers are listed in the following table.

**Table:** 

Genes	Primers
OCT4	F GTTGATCCTCGGACCTGGCTA
	R GGTTGCCTCTCACTCGGTTCT
SOX2	F TTTGTCGGAGACGGAGAAGC
	R TAACTGTCCATGCGCTGGTT
NANOG	F GTCTTCTGCTGAGATGCCTCACA
	R CTTCTGCGTCACACCATTGCTAT
GAPDH	F GAAAGCCTGCCGGTGACTAA
	R TTCCCGTTCTCAGCCTTGAC

### Immunocytochemistry

Treated and untreated adherent cells were incubated with 10 µg/ml Brefeldin A for 4 h before harvesting to block VEGFA secretion. Those cells as well as the tumorospheres were fixed in 4% paraformaldehyde followed by permeabilization in 0.1% Triton-X and blocking with BSA (3%). Cells were incubated with HIF1α and VEGFA antibodies (Thermo Fisher Scientific, USA) for 2 h followed by incubation with Alexa fluor 594 and Alexa fluor 488 tagged secondary antibodies (Thermo Fisher Scientific, USA) for 2 h. After that, cells were stained with DAPI. Images were captured using a fluorescence microscope (Olympus BX53, Japan) and LSM 510 Duo Scan laser scanning confocal microscope (Carl Zeiss, Germany).

### Sprout formation assay

Matrigel was thawed overnight and the plate was coated with Matrigel (BD Bioscience, USA) and incubated for 1 h for polymerization. HUVECs (1 × 10^4^) were seeded onto the Matrigel coated plate. After 8 h, media were changed with CM of interest. After 24 h of treatment tube formation was visualized microscopically and photographs were captured (DM 1000, Leica, Germany). The tube numbers were quantified by counting the branches between two discrete endothelial cells and length of the tubes were measured using Image J software by drawing a line along each tube and measuring the length in micrometer.

### Chick chorioallantoic membrane (CAM) assay

To investigate the role of CSC on angiogenesis, a modified chick chorioallantoic membrane assay was carried out. Briefly, 100 µl CM of treated and untreated spheres were mixed with 1.5 mg/ml type I collagen (Gibco, USA). 10 µl of the mixture were loaded on the piece of 13 mm Thermanox discs (Nunc, Denmark) and permitted to get polymerize. The disc was then applied to the chorioallantoic membrane of a 4 days-old embryo. After 72 h of incubation, the area around the loaded disc was photographed with a digital camera, and the numbers of newly formed vessels were counted by two observers in a double-blind manner (Min et al. 2004).

### Measurement of cellular ROS, NO and LPO level

Reactive oxygen species (ROS) level in the cells of treated and untreated CESs was measured by 2′,7′-dichlorofluorescein diacetate (DCFH-DA) standard protocol (Bhattacharjee et al. 2014). The production of nitric oxide (NO) were estimated by the level of stable NO metabolites, viz., nitrate (NO3^–^) and nitrite (NO2^–^) ions by reaction with Griess reagent (Bryan and Grisham 2007). Lipid peroxidation (LPO) were measured by estimating the formation of thiobarbituric acid reactive substances (TBARS) using TBA (0.8%) and expressed as nM TBARS formed/number of cells (Basu et al. 2016).

### Human Protein Atlas data, TNM plot data and receiver operating characteristic curve analysis

Human Protein Atlas (HPA) (https://www.proteinatlas.org/) database was used to explore the expressions of HIF1α and VEGFA in various types of cancers from large number of tissue samples of cancer patients (Thul and Lindskog 2018). TNM plot (https://tnmplot.com/analysis/) was used for analyzing HIF1α and VEGFA gene expression in various normal and tumor tissues of colon (Bartha and Győrffy 2622). Receiver Operating Characteristic Curve (ROC) plotter was used to compare the expression of HIF1α and VEGFA protein level in CRC tissues of patients who do not respond to 5FU based chemotherapy compared to the responders. Total 658 numbers of patients (379 responders and 279 non-responders) were included in this study (Özcan 2023).

### In silico analysis

Schrodinger software suite ver. 2020-3 (Schrödinger, LLC, New York, NY) was used for all docking and simulation run. HIF1α structure, 4ZPR was collected from protein data bank (RCSB PDB). PAS domain of HIF1α was isolated from 4ZPR. Structure was gone through H-bond minimization generating standard protonation state at pH-7.0 ± 2 and minimized using OPLS_2005 force field with restraint root mean square deviation (RMSD) cut off 0.30 Å (Jacobson et al. 2004). Receptor grid box was generated using Glide’s receptor grid generation module (Friesner et al. 2004). A cubic grid box of 20 Å × 20 Å × 20 Å was generated around the active residues for HIF1α. Molecular docking was performed to determine binding affinity of Orientin (PubChem CID-5281675) with the structure of HIF1α. The interaction of ligand with the residues of the spike was characterized by Glide docking score generated by extra precision (XP) docking (Friesner et al. 2004). Visualization of docked HIF1α structure with Orientin was done by Maestro 12.5 interface (Schrödinger, LLC, New York, NY) and was showing only the hydrogen bonds with their distances between the ligand atoms and the interacting receptor atoms. Desmond system builder was used for generating an orthorhombic simulation box of 10 Å × 10 Å × 10 Å around HIF1α-Orientin structure (Bhowmik et al. 2021; Bowers et al. 2006). A total 30 ns simulation was done for HIF1α-Orientin docked structure.

### Experimental animals and ethical issues

BALB/c Mice of 4–5 weeks age were received from the animal house of Chittaranjan National Cancer Institute. The animals were maintained at control temperature (23 ± 2 °C), humidity (55 ± 10%) and in alternating light and dark conditions (12:12, dark:light cycle). The mice were fed with standard pellet diet and water supplied ad libitum. All experiments were performed by following the CPCSEA guidelines with the approval of the animal ethics committee of Chittaranjan National Cancer Institute (CPCSEA Reg. No.−1774/GO/RBi/S/14/CPCSEA, India).

### Determination of toxicity of the compounds

To determine the toxicity of only Orientin treatment in various doses, the mice were divided into five groups. Each group contained six animals (n = 6). Each animal of the vehicle control group (VC) was left untreated, while, mice of other four groups were administered Orientin intravenously with 1 mg/kg b.w, 3 mg/kg b.w, 5 mg/kg b.w and 10 mg/kg b.w dosage respectively for 28 days. Another set of mice were divided in four different groups (n = 6); namely, 1) vehicle control group (VC), 2) Orientin treated group (treated with 5 mg/kg b.w/day Orientin intravenously), 3) 5FU treated group (treated with 5 mg/kg b.w 5FU intravenously in every alternate day) and 4) Orientin + 5FU treated group (treated with 5 mg/kg b.w Orientin intravenously every day and 5 mg/kg b.w 5FU intravenously in every alternate day). All the treatments were continued for 10 days. On day 11 all the mice were sacrificed and different parameters were studied.

### Development of colorectal tumor model

To set up therapeutic in vivo model, CT26 cells (2 × 10^6^ cells/animal), syngeneic to BALB/c mice were injected subcutaneously to the right hind limb of the each animal. Palpable tumor was formed after 10 days. Then the animals were randomly divided into four groups (n = 12) and treated accordingly: (1) Control group (Mice were left untreated for 10 days), (2) Orientin treated group (each animal was treated with 5 mg/kg b.w/day Orientin intravenously for 10 days), (3) 5FU treated group (each animal was treated with 5 mg/kg b.w 5FU intravenously on every alternate day for 10 days) and (4) Orientin + 5FU treated group (each animal was treated with 5 mg/kg b.w Orientin intravenously every day and 5 mg/kg b.w 5FU intravenously on every alternate day). Six mice from each group were sacrificed on day 11 and the others were left for performing survivability assay using Kaplan–Meier method (Hajra et al. 2018a).

### Measurement of cytotoxicity markers

Blood samples were collected from the retro-orbital venous plexus of mice for quantitative analysis of alanine aminotransferase (ALT), aspartate aminotransferase (AST), alkaline phosphatase (ALP), creatinine and blood urea nitrogen (BUN) in serum following kit protocol (Sigma Aldrich, USA). Optical densities were determined by using a spectrophotometer (Infinite® 200 PRO, TECAN, Switzerland).

### Estimation of ROS, NO, LPO and phase II detoxifying enzymes and reduced glutathione (GSH) level

The intracellular amounts of ROS, NO and LPO were measured in hepatic tissue homogenates of Orientin, 5FU or both treated mice as described previously (Bhattacharjee et al. 2014; Bryan and Grisham 2007; Basu et al. 2016). Glutathione-S-transferase (GST), superoxide dismutase (SOD), Catalase (CAT), Glutathione peroxidase (GPx) and reduced glutathione (GSH) activity in hepatic tissue were assessed as described previously (Hajra et al. 2018b). Values of all the parameters were calculated with respect to the amount of total protein present in liver homogenates.

### Histopathological analysis

Various organs and solid tumors collected after sacrificing the mice were embedded into paraffin after 10% neutral buffered formalin fixation. 4 μm sections were prepared and used for histopathological study. Standard Hematoxylin and Eosin (H&E) staining protocol was used and sections were visualized under light microscope (DM 1000, Leica, Germany) (Hajra et al. 2018a).

### Immunofluorescence assay

Immunofluorescence assay was carried out as described previously (Shimizu et al. 2023). Tissue sections were blocked with 3% BSA and incubated with primary antibodies like HIF1α, VEGFA, CD44 (Thermo Fisher Scientific, USA) and CD133 (Abcam, USA) at room temperature for 2 h. Alexa fluor 488 and Alexa fluor 594 tagged secondary (Thermo Fisher Scientific, USA) antibodies were added to all the tissue sections for 1 h followed by DAPI staining and images were visualized under fluorescence microscope (Olympus BX53, Japan).

### Statistical analysis

Statistical analysis were done by one way ANOVA followed by Tukey’s Multiple Comparison Test using GraphPad software and Microsoft office Excel 2010. All data were presented as mean ± SD. Significant difference was indicated when the p < 0.05.

## Results

### In vitro IC_50_ dose selection of Orientin and 5FU individually and in combination

**Fig. 1:**
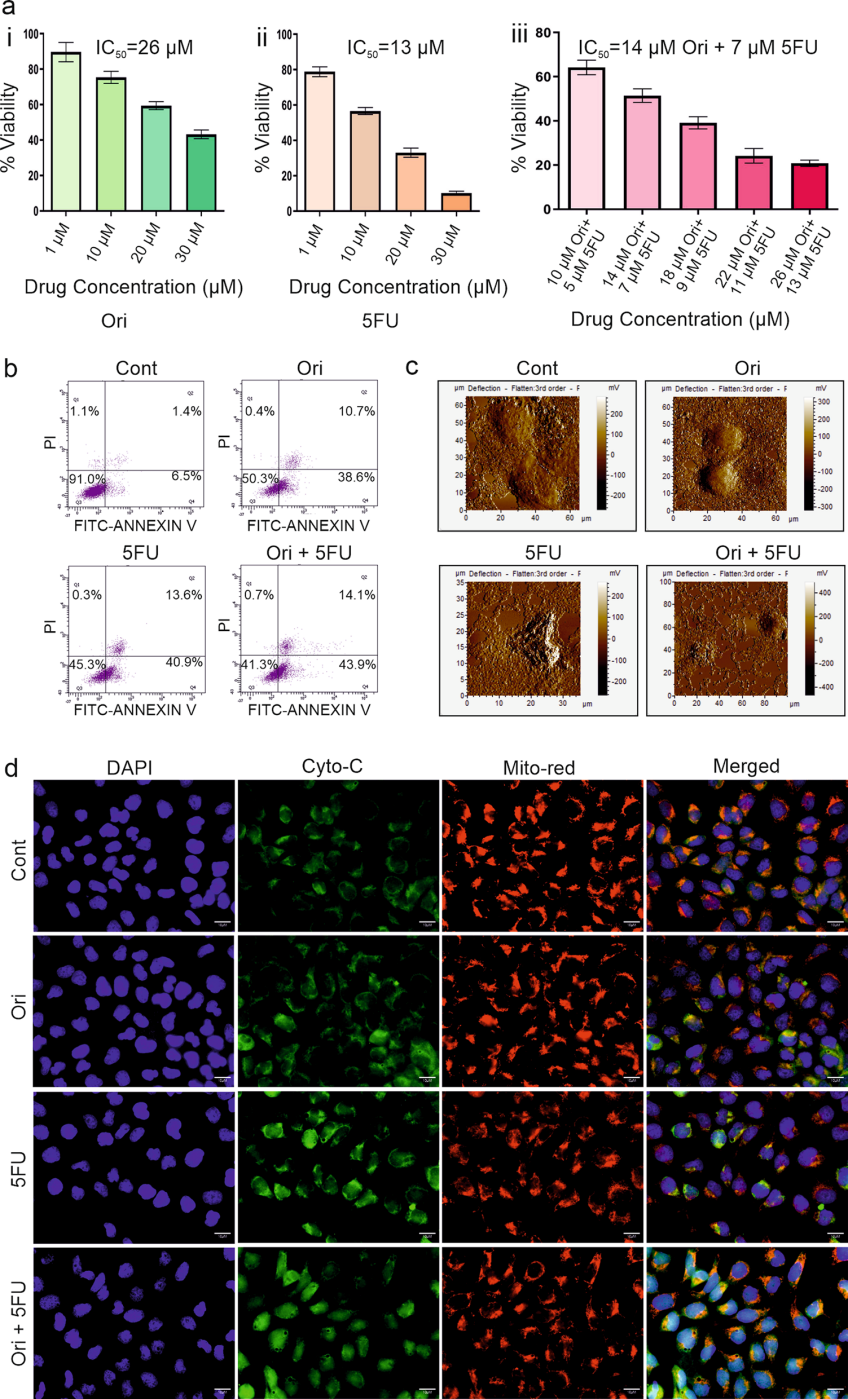
Antiproliferative and apoptotic effect of Orientin and 5FU alone or in combination. Antiproliferative effect of **a**) **i** Orientin (Ori) **ii** 5FU and **iii** Orientin and 5FU (Ori + 5FU) in combination are measured in HCT116 cell line. Cells are exposed to different dosages of these compounds for 48 h before MTT assays. The percentages of cell viability in various concentrations of the drugs represented by the bars and standard deviations (SD) are obtained from three independent experiments. **b** FACS analysis evaluates the percentages of apoptotic cell death by annexinV and PI staining of control, Orientin, 5FU and Orientin + 5FU treated HCT116 cells. **c** The morphological characteristics of control, 26 µM Orientin, 13 µM 5FU and 14 µM Orientin + 7 µM 5FU treated HCT116 cells are analysed by AFM. **d** Fluorescence micrographs of control and treated HCT116 cells show Cytochrome c release after 12 h treatment of Orientin, 5FU individually and both in combination. Cells are stained with anti-Cytochrome c antibody (green) (Cyto-c), mitochondria are stained with mitotracker red and nucleuses are stained with DAPI (blue)

The experimental doses for only Orientin and combination of Orientin and 5FU were determined by MTT assay in HCT116 cells after 48 h of treatment. The half-maximal inhibitory concentration (IC_50_) value for 5FU and Orientin were found to be 13 µM and 26 µM respectively (Fig. 1ai–ii) when used separately. The IC_50_ of 5FU and Orientin were reduced by approximately 50% for both the compounds (7 µM and 14 µM respectively) when used in combination (Fig. 1aiii), thereby suggesting that this combination therapy may reduce the adverse side effect of 5FU.These IC_50_ doses of Orientin and 5FU determined in case of monotherapy and combinatorial treatment were used to treat the cells for all the in vitro experiments.

### Combination of Orientin and 5FU induces apoptosis in lower doses compared to the monotherapy in CRC cells

Apoptosis is a natural process of programmed cell death to eliminate cells that have been damaged beyond repair. Apoptotic effect of Orientin, 5FU and combination of these drugs were verified by several in vitro assays using HCT116 cell line treated with the selected doses considering their respective IC_50_ doses. Flow cytometric analysis with Annexin V/PI staining after 48 h of treatment revealed that the percentage of apoptotic cells were increased to 54.5% and 49.3% when treated with 5FU and Orientin respectively for 48 h compared to the untreated cells (7.9%), whereas when used in combination the percentage of apoptotic cells were 58% (p < 0.0001) (Fig. 1b).

Furthermore, apoptotic effect of these drugs was also noticed by atomic force microscopy (AFM) after 24 h of treatment. As shown in Fig. 1c, conventional morphology with intact cell membrane was observed in untreated cells, but individual treatment with 5FU and Orientin caused cell shrinkage, distortion, nuclear condensation and fragmentation, membrane blebbing and morphological changes of cellular bodies (Fig. 1c). As expected, these effects were remarkably increased with emergence of apoptotic body and damaged pseudopodium structures for cell connections following combined use of the drugs suggesting enhanced efficacy compared to the individual treatments (Fig. 1c).

Early apoptotic event like cytochrome c release from mitochondria of HCT116 cells was also observed by fluorescent imaging after 12 h of treatment. Maximum amount of Cytochrome C was released in case of combination treated group compared to the untreated, or Orientin and 5FU monotherapy groups (Fig. 1d). These observations indicated that Orientin in combination with 5FU may induce apoptosis by intrinsic pathway.

### Orientin mitigates 5FU induced enrichment of CSC population when used as combinatorial treatment in vitro

**Fig. 2:**
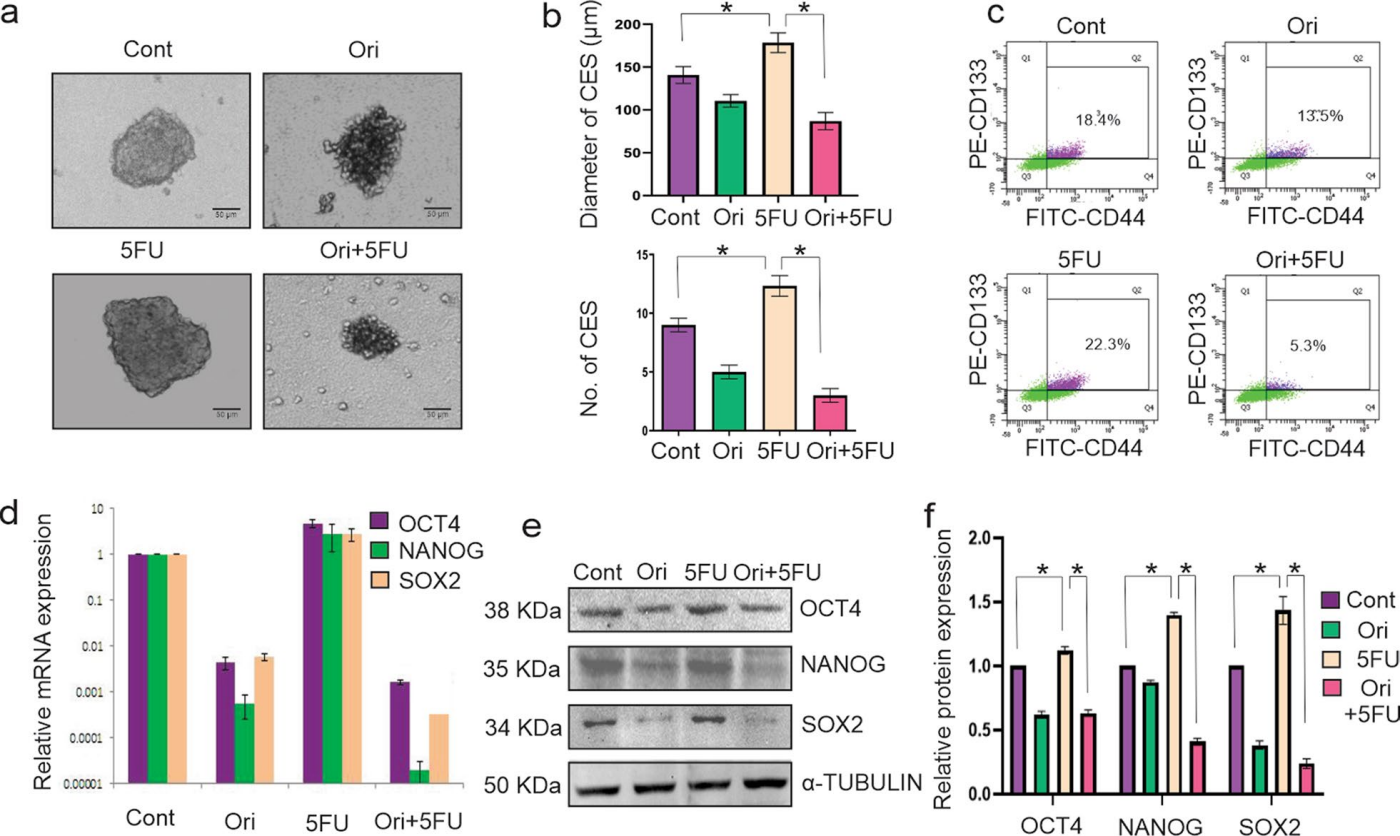
Inhibition of 5FU-induced HCT116-derived CSC enrichment by combinatorial treatment. **a** Representative images show CESs of control, 26 µM Orientin (Ori), 13 µM 5FU and 14 µM Orientin + 7 µM 5FU (Ori + 5FU) treated group after 24 h of treatment. **b** Diameter (upper panel) and number (lower panel) of spheres of each group are measured and represented in bar graphs with mean ± SD, n = 6, * indicates p < 0.01). **c** Expression profile of both CD44 (X axis) and CD133 (Y axis) in control, 26 µM Orientin. 13 µM 5FU and 14 µM Orientin + 7 µM 5FU treated spheres are characterized by flow cytometry. Cell populations are identified based on PE-conjugated anti-CD133 and FITC-conjugated anti-CD44 antibodies, where percentages of double positive cell population are represented in the upper right quadrate of each plot. **d** Total RNA extracted from sorted CD44^+^/CD133^+^ cell population of control, 26 µM Orientin, 13 µM 5FU and 14 µM Orientin + 7 µM 5FU treated group are analyzed by qRT-PCR for OCT4, NANOG and SOX2. **e** Western Blot analysis represents the expression of OCT4, NANOG and SOX2 in sorted CD44^+^/CD133^+^ cell population of control, 26 µM Orientin. 13 µM 5FU and 14 µM Orientin + 7 µM 5FU treated group. **f** Graph represents densitometric value of the bands of OCT4, NANOG and SOX2 in control and different treatment groups

CSCs have attracted research interest as they are responsible for development of drug resistance and cancer relapse. To determine the CSCs inhibitory effect of combinatorial treatment, we developed tumorospheres from HCT116 cells to enrich CSCs where we found 29-fold increase of CD44^+^/CD133^+^ cells compared to the non tumorospheres conditions (p < 0.0001) (Fig. S1). Treatment of tumorospheres with 5FU significantly increased diameter (p = 0.0064) and number of spheres (p = 0.0314) compared to the control group (Fig. 2a, b). In contrast, significant decreases in both the parameters were observed when the spheres were treated with Orientin alone (p = 0.0001 for diameter and p = 0.0002 for number of spheres) or in combination with 5FU (p < 0.0001) compared to the only 5FU treated group (Fig. 2a, b). Besides, flow cytometric analysis showed that 5FU treatment increased number of CD44^+^/CD133^+^ cell population to 22.3% compared to the untreated group (18.4%) inside the CESs (p < 0.0001), suggesting that 5FU treatment caused enrichment of CSCs. On the other hand, Orientin alone and combinatorial treatment decreased percentage of this cell population to 13.5% and 5.3% respectively (p < 0.0001) (Fig. 2c).

To further determine the stemness properties of CSCs in tumorospheres, CD44^+^/CD133^+^ cell population were isolated by MACS column-based cell sorting methods after 24 h of treatment with 5FU, Orientin and combination of both. 5FU treatment increased mRNA level of various stemness maintenance factors, whereas, Orientin alone and in combination with 5FU decreased those mRNA levels (p < 0.0001) (Fig. 2d).

These findings were further validated by determining protein expression level by western blotting analysis where Orientin alone or in combination with 5FU downregulated OCT4, NANOG and SOX2 compared to the only 5FU treated group (p < 0.0001) (Fig. 2f, g). Hence, it is evident from these data that 5FU treatment enriched CSCs population in tumour, whereas Orientin in combination with 5FU restricted CSC enrichment as well as reduced the stemness properties of CSCs.

### Combinatorial treatment of Orientin and 5FU inhibits CSC mediated angiogenesis ex vivo

**Fig. 3:**
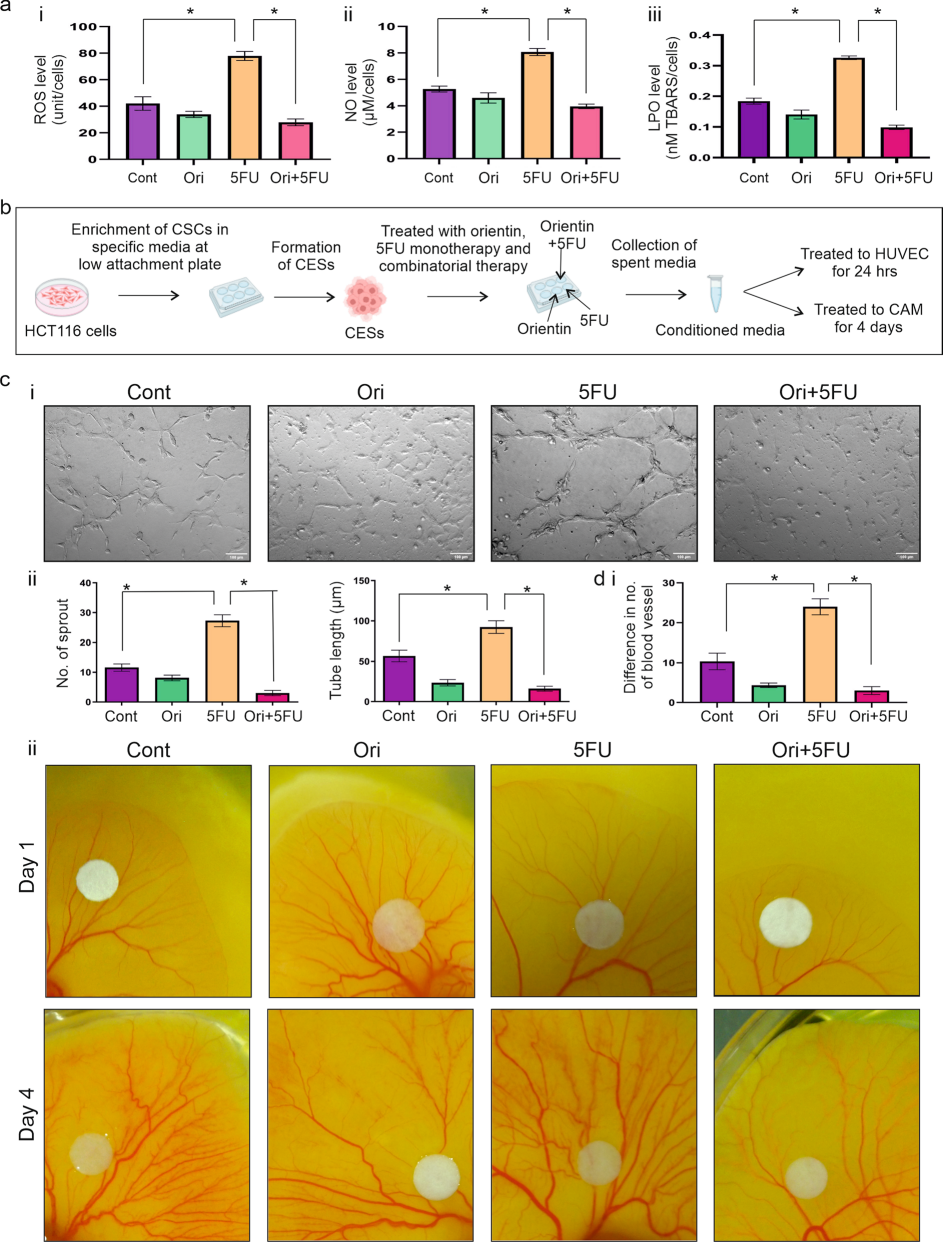
Inhibitory effect of combinatorial treatment on 5FU induced CSC-mediated angiogenesis. **a** Graphs represent **i** ROS, **ii**) NO and **iii** LPO level in 5 million cells of control CES and CES treated with 26 µM Orientin (Ori), 13 µM 5FU and 14 µM Orientin + 7 µM 5FU (Ori + 5FU) for 24 h. Bar diagram shows mean ± SD value with * indicating p < 0.01. **b** Graphical illustration of treatment procedure **c**) **i** Sprout formation by HUVEC cells after 24 h of incubation with CM of control CES, 26 µM Orientin treated CES, 13 µM 5FU treated CES and both 7 µM 5FU and 14 µM Orientin treated CES are photographed under bright field microscope. **ii** Graphs represent number of sprouts and tube length in bar diagram with mean ± SD (n = 6, * indicates p < 0.01). **d** CM of control CES and 26 µM Orientin, 13 µM 5FU and both 7 µM 5FU & 14 µM Orientin treated CESs are mixed with type I collagen gel and loaded on the CAMs of four days old chick embryos. **i** Graph represents difference in number of blood vessels between day 4 and day 1 of each group (n = 6, * indicates p < 0.01). **ii** Representative images of the CAMs of each group acquired on day1 and day 4 after incubation with CMs of untreated and treated CESs

It was found that while 5FU treatment significantly (p < 0.0001) increased ROS, NO and LPO production in tumorospheres compared to the control group, a significant (p < 0.0001) decrease was noticed on their production after treatment with Orientin alone and in combination with 5FU (Fig. 3ai–iii). As these parameters are associated with angiogenesis, we performed sprout formation assay and CAM assay to evaluate anti-angiogenic potentialities of combinatorial treatment. The treatment procedure is graphically illustrated in Fig. 3b. HUVEC exposed to CM of 5FU treated tumorospheres significantly (p < 0.0001) instigated sprout formations by increasing the numbers and length of tubes compared to the cells treated with CM of control CESs (Fig. 3ci–ii). In contrast, CM of Orientin treated CESs remarkably reduced (p < 0.0001) the numbers and tube length of HUVEC compared to the HUVEC treated with CM of 5FU treated CESs (Fig. 3ci-ii). Moreover, maximum reduction in sprout formation and tube length was observed when HUVEC were exposed to CM of Orientin and 5FU treated tumorospheres (p < 0.0001) (Fig. 3ci–ii).

These findings were again validated by the results of CAM assay where we found significant (p < 0.0001) increase in number of blood vessel formation when exposed to the CM of 5FU treated tumorospheres on day 4 in comparison to day 1 (Fig. 3di–ii). Non-significant change was observed when CAM was treated with CM of Orientin treated CESs alone (Fig. 3di–ii). We also found significant (p < 0.0001) inhibition of blood vessel formation in CAM exposed to CM of combination treated CESs in comparison to 5FU treated CESs (Fig. 3di–ii). Therefore, these findings suggested that combination of Orientin and 5FU has potential to inhibit 5FU induced CSC-mediated angiogenesis.

### Databases showed that HIF1α and VEGFA are overexpressed in CRC patients

One of the most important proteins involved in angiogenic progression is HIF1α which plays a significant role in CSC proliferation. According to the Human Protein Atlas (HPA) database, HIF1α has the highest expression in CRC among different cancers (Fig. S2a). Along with this, VEGFA, another crucial angiogenic factor also shows the same pattern of expression (Fig. S2a). However, from TNM database it is evident that expression of HIF1α and VEGFA is significantly (p = 3.48e^−04^ and 1.23e^−113^) higher in colorectal tumor tissues compared to the normal tissues (Fig. S2b).

As the previous findings provide information on the induction of CSC-mediated angiogenesis by 5FU treatment, it was of interest to examine the expression level of HIF1α and VEGFA in CRC patients receiving chemotherapy with 5FU. Further, differential expression of these genes in 5FU treated CRC patients (379 responders and 279 non-responders) were validated by Receiver Operating Characteristic Curve (ROC) plotter. Both HIF1α and VEGFA were upregulated in CRC patients, who do not respond to 5FU (Fig. S2c). The ROC analysis of these genes indicates that in CRC HIF1α and VEGFA could be a prominent target for regulation of angiogenesis. The results also indicate a positive correlation between the resistances to 5FU based chemotherapy and the upregulation of these genes.

### In silico study determines that Orientin inhibit HIF1α by binding at its PAS domain

**Fig. 4:**
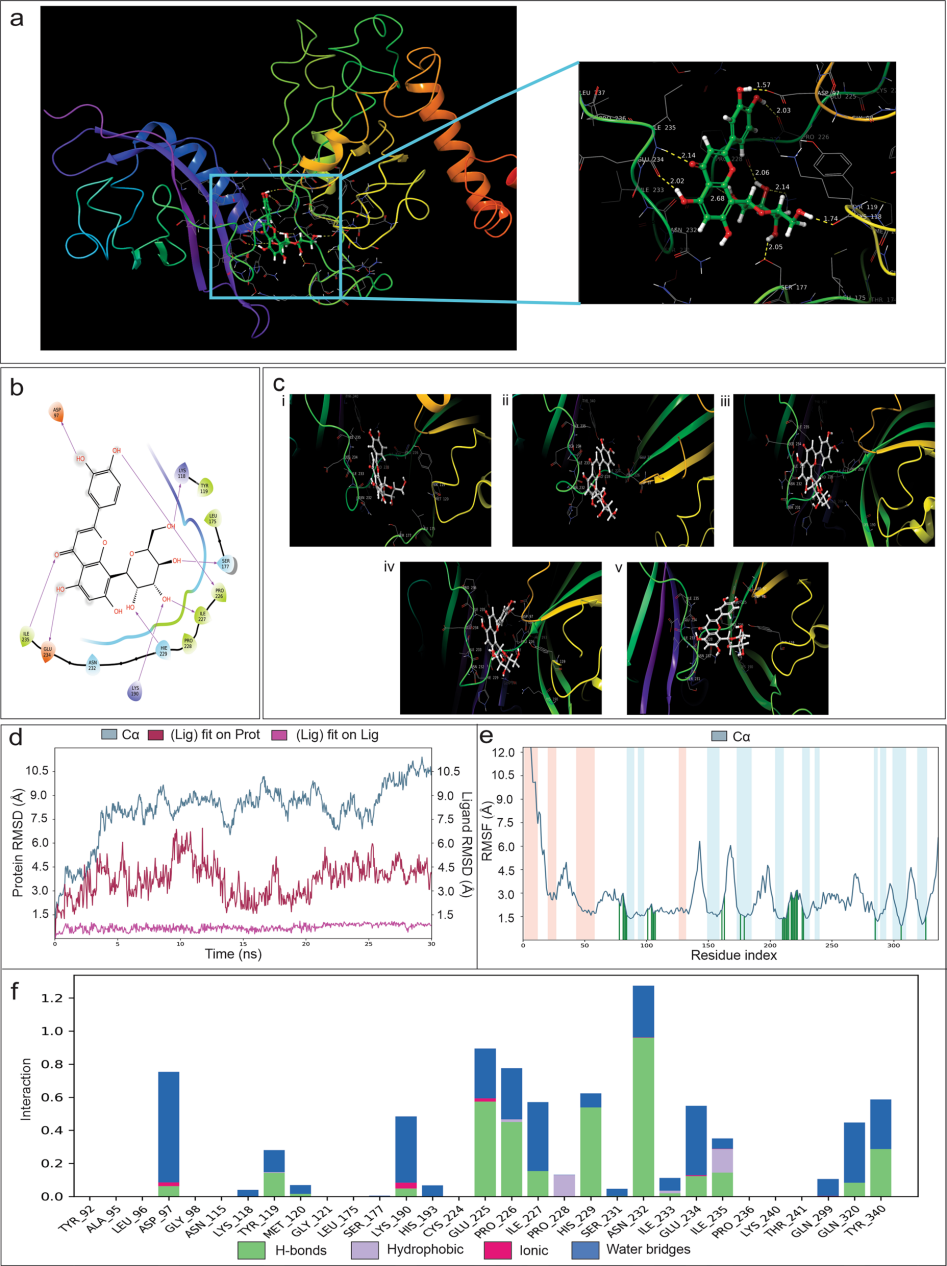
Validation of binding of Orientin with HIF1α in silico. **a** Figure represents binding of Orientin to the 3D structure of HIF1α (PDB id: 4ZPR). Inset shows that Orientin binds at the PAS domain of this protein. **b** Figure represents major interacting residues of HIF1α with Orientin. **c** MD simulation shows the binding of Orientin to the PAS domain of this protein at I) 0 ns, II) 10 ns, III) 15 ns, IV) 20 ns and V) 30 ns. **d** Graph represents overall structural changes (RMSD) in the Orientin bound HIF1α over 30 ns time. **e** Graph shows root mean square fluctuations (RMSF) in the backbone and side chains of HIF1α. Ligand contacts are shown in green lines. **f** Bar graph shows interaction fractions of different types of interactions in Orientin- HIF1α complex

To assess whether Orientin has potential to inhibit HIF1α activity by binding with it, an in silico docking was performed. Figure 4a represents the structure of human HIF1α (PDB id: 4ZPR) which serves as the template for the development of in silico model. The enlarged inset shows the best possible docked structure of Orientin and HIF1α. According to this structure, Orientin interacted with PAS domain of HIF1α. This domain is responsible for heterodimerization of HIF1α, which in turn, helps in the formation of the active form of this transcription factor. Therefore, inhibiting this domain by any molecule can result in loss of activity of this protein (Park et al. 2006). According to the MD simulation data, Orientin formed Hydrogen bond with various amino acids of HIF1α like ASP97, LYS118, TYR119, LEU175, SER177, PRO226, ILE227, PRO228, HIE229, LYS190, ASN232, GLU234 and ILE235 (Fig. 4b). Figure 4c represents the docked conformations of Orientin with HIF1α. Confirmations were recorded during the MD simulation in various time points such as 0 ns, 10 ns, 15 ns, 20 ns and 30 ns (Fig. 4ci–4cv). Orientin formed a stable binding with its target protein during the uninterrupted simulation of 30 ns (Video S1). The average Root Mean Square Deviation (RMSD) value observed during the simulation was 4.5 Å. The RMSD value of the ligand with respect to the ligand was low enough, which suggests that structure of Orientin remained unaltered during the simulation. Additionally, RMSD plot of ligand with respect to protein shows the change of the compound orientation in the binding site with time. However, the overall conformation became stable after 5 ns (Fig. 4d). Figure 4e represents the low fluctuation of the amino acid residues of the protein, which indicates a stable docked conformation between the protein and the ligand. Moreover, the highest number of interactions was observed between the ASN 232 residue and the protein during the whole simulation (Fig. 4f). Although, Orientin formed hydrogen bonds, water bridges, ionic and hydrophobic interactions with HIF1α, major interactions occurred via formation of hydrogen bonds (Fig. 4f). Cumulatively, all of these data suggest that Orientin forms a stable binding in the PAS domain of HIF1α.

### Combination of Orientin and 5FU attenuates CSC-mediated angiogenesis by inhibiting expression of HIF1α and VEGFA

**Fig. 5:**
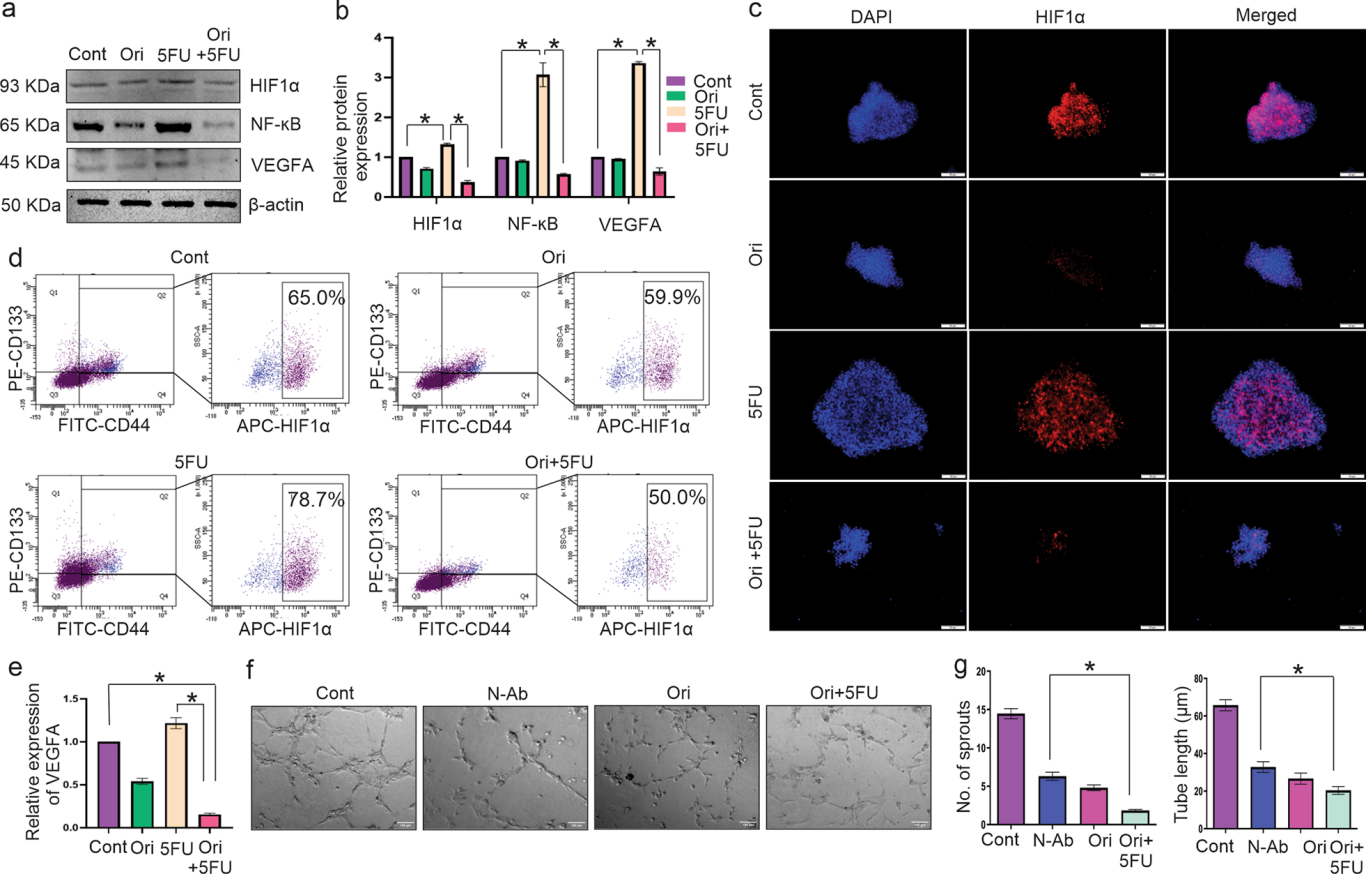
Downregulation of 5FU induced HIF1α and VEGFA expression by combinatorial treatment in vitro in CESs. **a** Figure represents the expression of HIF1α, NF-κB and VEGFA protein in untreated CESs and CESs after 24 h treatment of 26 µM Orientin (Ori), 13 µM 5FU and 14 µM Orientin + 7 µM 5FU (Ori + 5FU). **b** Densitometric value of each western blot is represented graphically in the Figure (n = 3). **c** Fluorescence imaging confirms upregulation of HIF1α (red) in control and 5FU treated group, whereas Orientin and combinatorial treatment reduces the expression of this protein. Nucleuses are stained with DAPI (blue). Merged images show the localization of HIF1α mainly in the core of the spheres. **d** Flow cytometry analysis represents percentage of HIF1α expressing cells within the CD44^+^/CD133^+^cell population of control, Orientin, 5FU and both treated CESs. Cell population double stained with anti-CD44-FITC and anti-CD133-PE are represented in upper right quadrate of the plots (left panel). Percentages of HIF1α-APC^+^ cells within CD44^+^/CD133^+^ CSC population are mentioned in each plot. **e** Bar diagram with SD represents relative expression of VEGFA measured in ng/ml secreted by control CESs and CESs upon Orientin, 5FU and combinatorial treatment analyzed by ELISA with respect to control group (n = 3, * indicates p < 0.001). **f** Sprout formation is analyzed in HUVECs treated with CM of control CESs, CM of 26 µM Orientin treated CESs, CM of 5 µg/ml VEGFA neutralizing antibody treated CESs (N-Ab) and CM of CESs treated with 26 µM Orientin and 13 µM 5FU in combination (Ori + 5FU). **g** Number of sprouts and tube length of HUVECs are quantified and represented in bar diagram (n = 3, *indicates p < 0.01)

Expression of HIF1α and VEGFA in CES were analyzed by immunoblotting assay. 5FU treatment upregulated HIF1α and VEGFA expression compared to the without treatment group, whereas, in case of combinatorial treatment HIF1α and VEGFA expression were significantly (p < 0.0001) downregulated (Fig. 5a-b). To explore how Orientin downregulated expression of HIF1α, we determined expression of NF-ĸB in CES by Western blot analysis. Our results showed that NF-ĸB were significantly (p < 0.0001) downregulated by Orientin alone and combination of Orientin with 5FU, whereas overexpression of NF-ĸB was noticed in 5FU treated group (Fig. 5a, b). Expression of HIF1α within CESs was further validated by immunocytochemical analysis, which again showed the similar pattern like the previous result (Fig. 5c).

As CES contains heterogeneous population, we analyzed percentage of HIF1α positive cells within CD44^+^/CD133^+^ cell population in CES by FACS analysis. Our results showed that 5FU treatment increased more than 20% HIF1α positive cells in comparison to without treatment group, whereas, almost 30% reduction of HIF1α positive cells were recorded after combinatorial treatment compared to only 5FU treated group (p < 0.0001) (Fig. 5d). The amount of VEGFA secreted into the CM of CES was measured by ELISA test. 5FU stimulated the secretion of VEGFA compared to the control group, whereas, VEGFA level was significantly reduced (p < 0.0001) in CM of combinatorially treated group (Fig. 5e).

To evaluate whether Orientin attenuates angiogenesis by inhibiting VEGFA activity or not, we compared its effects with known VEGFA neutralizing antibody on HUVEC cells. As expected, CM of CES treated with VEGFA neutralizing antibody showed a significant attenuation in the number of sprouting (p < 0.0001) as well as length of tubes formation (p < 0.0001) compared to the control group. CM of Orientin treated CES also showed same level of inhibition in the sprouting of HUVEC cells whereas CM of combinatorially treated CES showed more suppressive effect of sprouting in HUVEC compared to VEGFA neutralizing antibody treated CES (Fig. 5f-g). Therefore, these findings suggest that Orientin is capable of inhibiting VEGFA activity like its neutralizing antibody, which advocates its efficacy against 5FU induced HIF1α mediated VEGFA overexpression in CES.

### Orientin alone and in combination with 5FU inhibits VEGFA by downregulating HIF1α and reduces enrichment of CSCs formed by HIF1α overexpressed cells

**Fig. 6:**
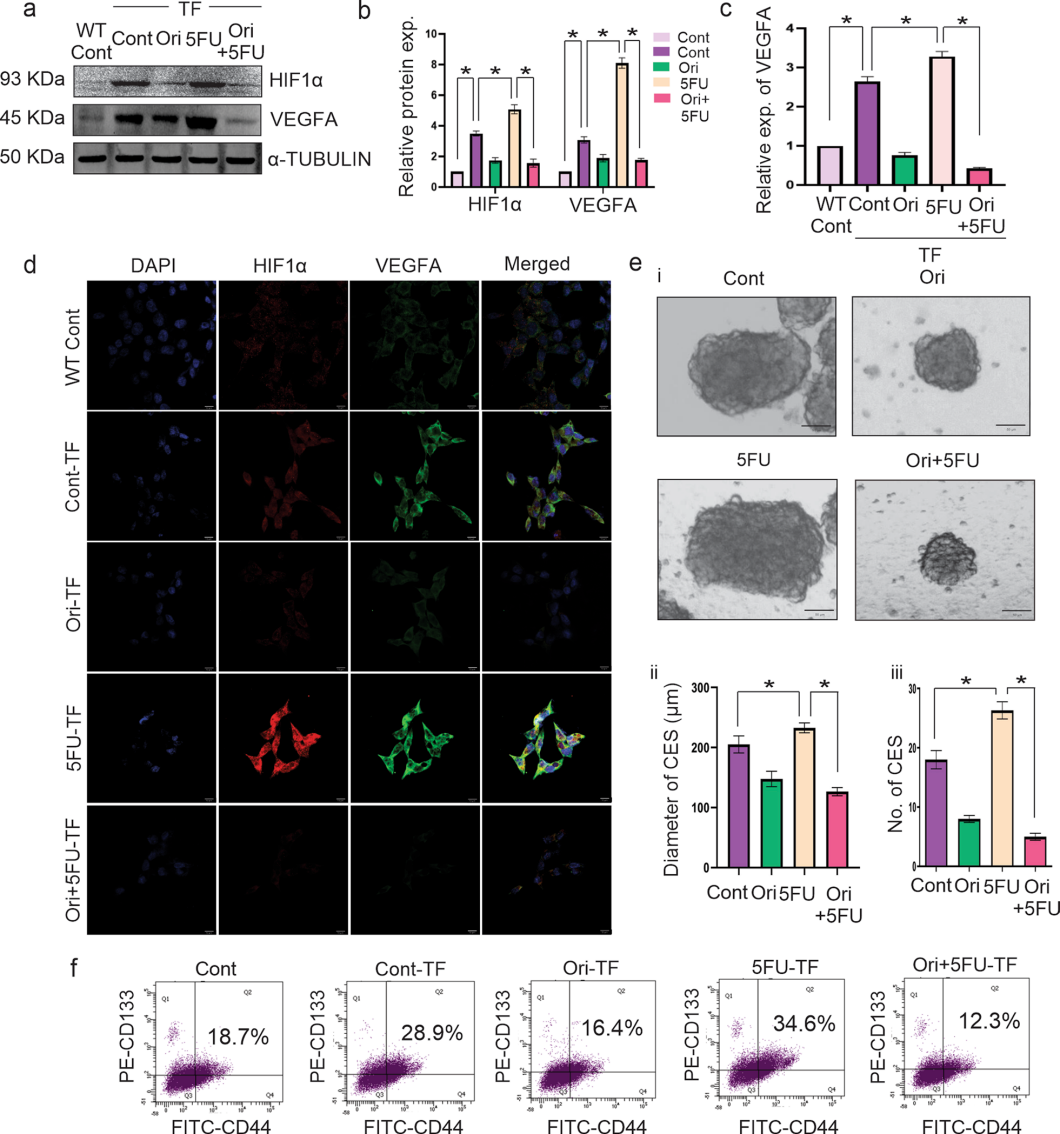
Reduction of 5FU induced HIF1α and VEGFA expression by combinatorial treatment in vitro in HIF1α transfected CRC cells. **a** Expression of HIF1α, and VEGFA in non-transfected (WT Cont) and HIF1α transfected control (Cont), Orientin (Ori), 5FU and Orientin + 5FU (Ori + 5FU) treated groups are analyzed by western blotting. **b** Densitometric values of each band are mentioned in this Figure. **c** Bar diagram with SD represents the relative expression of VEGFA (measured in ng/ml) secreted from non-transfected (N.TF) and transfected cells of control and treated groups measured by ELISA. **d** Representative immunofluorescence images show the expression of HIF1α (red), VEGFA (green), and DAPI (blue) in non-transfected control, transfected control (Cont-TF) and Orientin treated (Ori-TF), 5FU treated (5FU-TF) and Orientin + 5FU treated (Ori + 5FU-TF) transfected HCT116 cells. **e** CESs are generated from control and differently treated transfected cells. **i** Images of the spheres captured after 7 days under light microscope. **ii** Graphs represent diameter and **iii** numbers of those spheres with mean ± SD, n = 3, * indicates p < 0.01. **f** CESs formed from non-transfected control (WT Cont) and transfected control (Cont-TF) and transfected treated cells are stained with FITC-CD44 (plotted in X axis) and PE-CD133 antibodies (plotted in Y axis). Percentage of CD44^+^/CD133^+^ cells are analyzed by flow cytometry and represented in the upper right quadrate of each plot

To provide further evidences that Orientin downregulates VEGFA expression by inhibiting HIF1α, we transfected HCT116 cells with plasmid encoding HIF1α. We found more than three-fold upregulation of HIF1α (p < 0.0001) expression compared to the non-transfected cells. After 5FU treatment, expression of HIF1α increased approximately five-fold (p < 0.0001) compared to non-transfected cells but the effect was almost similar to normal after Orientin and 5FU combinatorial treatment. As expected, with the over-expression of HIF1α, VEGFA expression was also altered in the same pattern (Fig. 6a, b).

In addition to this, quantitation of VEGFA secretion was studied by ELISA test. Our results indicated that VEGFA was secreted more than three-fold (p < 0.0001) in HIF1α over-expressed condition compared to wild type cancer cells, whereas after combinatorial treatment it was back to normal (Fig. 6c). Immunocytochemical analysis also demonstrated the upregulation of HIF1α and VEGFA in transfected cells compared to non-transfected cells. After 5FU treatment, the expression of both the proteins were more prominent in transfected cells, whereas Orientin individually or in combination with 5FU reduced these protein expressions (Fig. 6d) which suggests a strong positive correlation between HIF1α and VEGFA in CRC cells.

Additionally, HIF1α transfected cells have potential to develop CESs with increased diameter (p < 0.0001) and number (p < 0.0001) compared to non-transfected cells. 5FU treatment significantly (p < 0.0001) increases both of those parameters in CESs, whereas Orientin alone and in combination with 5FU can decrease diameter and number of spheres (p < 0.0001) (Fig. ei-iii). Flow cytometric analysis showed that HIF1α transfected cells have better potential to enrich CSC population (28.9%) within the tumorospherers compared to the non-transfected HCT116 cells (18.7%) (p < 0.0001) (Fig. 6f). On the other hand, the percentage of CD44^+^/CD133^+^ cell population within the transfected cells derived tumorospheres decreased to 16.4% after Orientin treatment (p < 0.0001). Contrastingly, 5FU increased (p < 0.0001) this CSC population to 34.6%, whereas Orientin in combination with 5FU displayed the highest efficacy (p < 0.0001) to reduce this cell population to 12.3% (Fig. 6f). Taken together, these data confirm that Orientin inhibits the expression of HIF1α, resulting in the attenuation of enrichment of CSCs along with the CSC-mediated angiogenesis induced by 5FU in vitro. Further in vivo validation of these findings is required.

### Orientin can mitigate hepatotoxicity and nephrotoxicity induced by 5FU in vivo

Before determining if Orientin can mitigate toxic effect of 5FU we sought to find out whether Orientin itself induces any toxicity in vivo. Administration of 5 mg/kg b.w Orientin significantly decreased (p < 0.0001) LPO level compared to the control group (Fig. S3ai). Additionally, increase in expression of GSH (p < 0.0001) and activity of different hepatic enzymes such as GST, GPx, SOD and CAT (p < 0.0001) after 5 mg/kg b.w Orientin treatment further proved its nontoxicity (Fig. S3aii-vi). Moreover, no histopathological changes of liver, heart, lung and kidney tissues in 5 mg/kg b.w Orientin treated group were found compared to the control group (Fig. S3bi–iv). We also found that different toxicity parameters display almost similar value after 10 mg/kg b.w and 5 mg/kg b.w Orientin treatment. These findings drive us to select the dose 5 mg/kg b.w for further in vivo experiments.

5FU is one of the prototype toxicity-inducing conventional chemotherapeutic agents which may exert hepatotoxicity or/ and nephrotoxicity. Therefore, a novel therapeutic approach to restrict 5FU induced toxicity is highly needed. To evaluate if Orientin has any cytoprotective effect, we studied the expressions and activities of different phase II detoxifying enzymes in hepatic tissues of BALB/c mice treated with 5FU, Orientin and combination of 5FU and Orientin for 10 days (Fig. S4). 5FU treatment significantly (p < 0.0001) reduced the expression of GSH and activity of various antioxidant enzymes like GST, GPx, SOD and CAT compared to the vehicle control group in hepatic tissues (Fig. S4i-S4v). In contrast, significant ((p < 0.0001) increases in these parameters were observed in BALB/c mice when treated with Orientin and 5FU in combination (Fig. S4i-S4v) compared to the 5FU treated group. Besides, 5FU was found to significantly ((p < 0.0001) induce LPO, ROS and NO in hepatic tissues compared to the control group but after treatment with Orientin and 5FU these values significantly decreased near to normal (Fig. S4vi–S4viii). Histopathological analysis showed that hepatic tissue architecture remained normal in Orientin treated mice (Fig. S5a). Contrastingly, distorted central vein, necrosis, cellular and blood infiltration occurred in the hepatic tissue of 5FU treated mice, whereas morphology of liver tissue was normal when mice were treated with Orientin and 5FU in combination (Fig. S5a). Similarly, Orientin alone and in combination with 5FU did not induce any toxicity in kidney tissue (Fig. S5b). In contrast distorted Bowman’s capsule, glomeruli, blood and cellular infiltration were found in renal tissue of 5FU treated group (Fig. S5b).

In addition, our results also showed that other hepatotoxicity markers like serum Alanine Aminotransferase (ALT), Aspartate Aminotransferase (AST) and Alkaline Phosphatase (ALP) were also increased after 5FU treatment (Table 1). However, contrasting effect was noticed in case of combinatorial treatment (Table 1). Moreover, 5FU also created nephrotoxicity by increasing Blood Urea Nitrogen (BUN) and creatinine in kidney tissues (Table 1). These parameters remained normal when BALB/c mice were administered 5FU with Orientin (Table 1).

**Table 1:** Effect of Orientin, 5FU and their combination on serum ALT, AST, ALP, BUN, creatinine levels in BALB/c mice

Groups	ALT (U/ml)	AST (U/ml)	ALP (U/ml)	BUN (mg/dl)	Creatinine (mg/dl)
Vehicle Control	54.62 ± 2.45	134.64 ± 2.91	101.89 ± 2.90	22.35 ± 0.36	0.55 ± 0.022
Orientin	49.08 ± 1.99	134.7 ± 2.00	94.23 ± 2.10	21.48 ± 0.53	0.49 ± 0.020
5FU	121.70 ± 3.95^*^	219.16 ± 2.83^*^	168.44 ± 4.09^*^	30.76 ± 0.46^*^	0.73 ± 0.023^*^
Orientin + 5FU	64.67 ± 2.94^#^	150.92 ± 2.57^#^	114.41 ± 3.15^#^	24.83 ± 0.50^#^	0.58 ± 0.022^#^

Data are represented as mean ± SD, n = 6 in each group. *P < 0.05 significantly different from vehicle control. ^#^P < 0.05 significantly different from 5FU. (one-way ANOVA followed by Tukey’s multiple comparison test)

### Combination of Orientin and 5FU displays anti-tumor efficacy and significantly reduces CSC-mediated angiogenesis in tumor-bearing mice

**Fig. 7:**
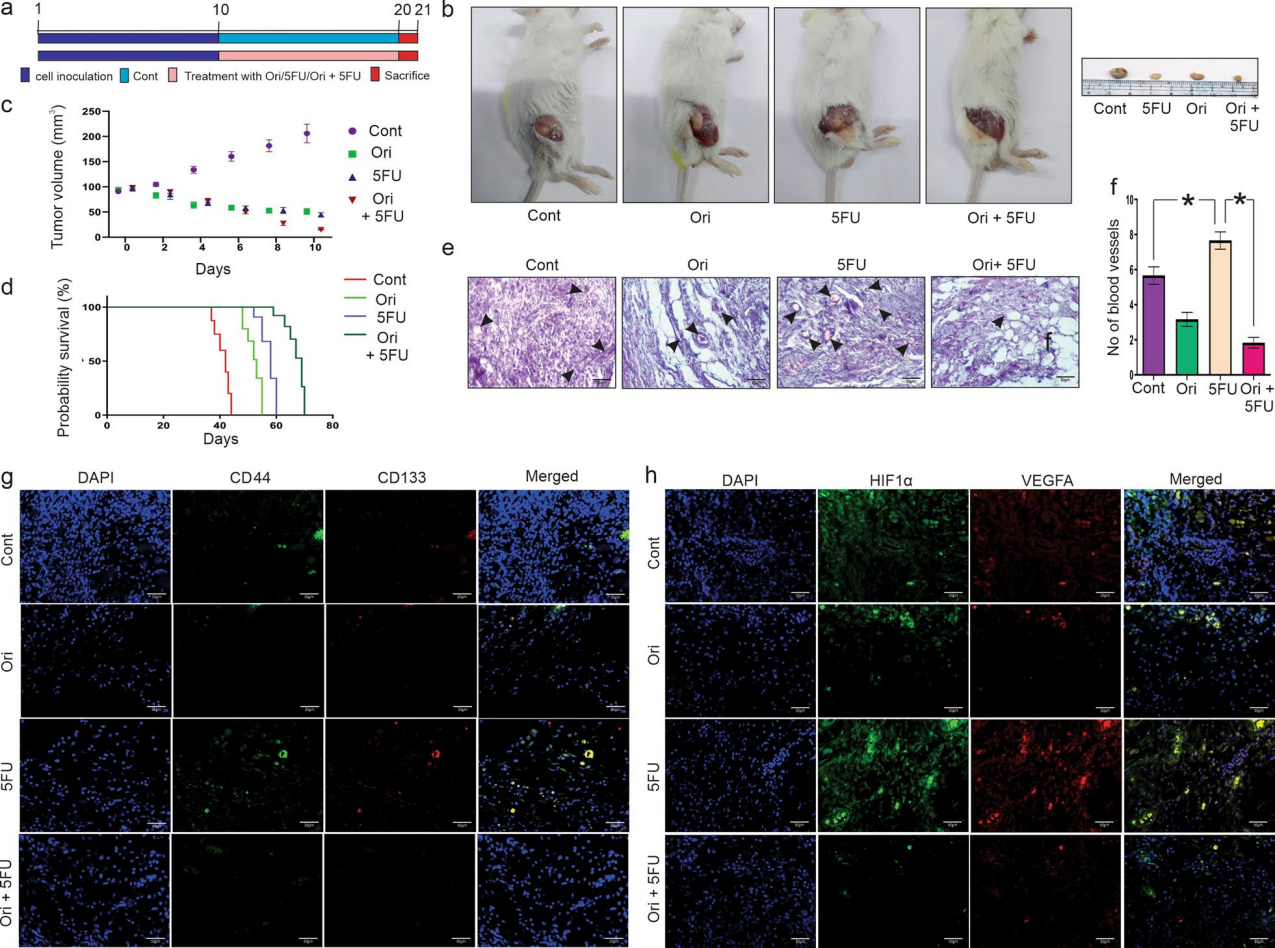
Ablation of CSC enrichment and angiogenesis in tumors of mice treated with combination of 5FU and Orientin. **a** Experimental design to study the effect of Orientin (Ori), 5FU and Orientin + 5FU (Ori + 5FU) on CT26 cell induced solid tumor is illustrated schematically. **b** Images display the tumors after 10 days of Orientin, 5FU and Orientin + 5FU treatment in combination. **c** Tumor growth curve after intravenous administration of Orientin, 5FU and both in combination for 10 days is represented in the graph. Volume of the tumors are measured in every alternate day during treatment schedule and represented as mean ± SD. **d** Graph shows effect of Orientin, 5FU and both in combination on survivability of solid tumor bearing mice. Data are analyzed using Kaplan–Meier method. Survival time plotted as days after tumor transplantation. **e** Histopathological analysis shows the morphology of tumor tissue sections of mice from control, Orientin, 5FU and Orientin + 5FU group (H&E staining). All the images are captured under bright field microscope with ×40 magnification. Blood vessels shown in the images are marked with black arrows. **f** Number of blood vessels in tissue sections are counted and represented in the bar diagram with mean ± SD (n = 6, * indicates p < 0.05). **g** Immunofluorescence images of tumor tissues from mice of different groups represent the expression of CD44 (green) and CD133 (red). Tissue sections are counterstained with DAPI (blue) and the merged images shows expression of CD44^+^/CD133^+^ cells (yellow). **h** Tumor tissue sections of different groups of mice are stained with HIF1α (green) and VEGFA (red). Nucleuses have taken colour of DAPI (blue)

To determine the effect of Orientin in CSC-mediated angiogenesis in vivo, CT26 colon cancer cell-induced tumor model was developed. The tumor bearing mice were treated with Orientin, 5FU and both in combination for 10 days as described in ‘Materials and Methods’ section. A schematic representation of this treatment schedule is illustrated in Fig. 7a. Along with the anti-angiogenic effect, the in vitro findings indicated that Orientin alone and in combination with 5FU have induced apoptosis within cancer cells. In vivo results also showed a significant (p < 0.0001) decrease in the tumor volume by all of these treatments compared to the control group, suggesting significant anti-tumor efficacy of these compounds. Although, no significant differences was observed in the tumor size of Orientin and 5FU treated groups, combinatorial treatment was found to have the highest efficacy in inhibiting tumor progression (Fig. 7b, 7c). Significant (p < 0.0001) increased life span was also noticed in all the treatment groups compared to the control group. The Kaplan Meier’s survival plot shows that administration of Orientin and 5FU both in combination led to the highest increase (p < 0.0001) of survival rate compared to the control and 5FU treated group (Fig. 7d). The anti-tumor efficacies of these drugs were further evaluated by histopathological analysis of the tumor tissues. Tissues of untreated mice displayed sheets of malignant cells along with higher cell density, but tumor tissues lost their compactness in Orientin treated group. In case of 5FU treatment, cell numbers were found to be reduced. The combinatorial treatment produced the best anti-tumor effect reflected as displaying the least density of tumor cells with hyperchromatic and pyknotic nuclei in tumor tissues (Fig. 7e). Although 5FU treatment causes reduction in tumor volume, it encouraged tumor angiogenesis by increasing blood vessels number (p < 0.0001), but in Orientin treated group reduced (p < 0.0001) blood vessels were observed (Fig. 7e -f). Combinatorial treatment showed the highest anti-angiogenic efficacy compared to the other groups. This is consistent with our in vitro and ex vivo findings.

Fluorescence imaging showed that 5FU increased both CD44 and CD133 expressing cells in the tumor tissues of 5FU treated mice suggesting that this chemotherapeutic drug encourages CSC enrichment. Contrastingly, Orientin treatment caused reduction in the CD44^+^/CD133^+^ cells suggesting presence of a smaller number of CSCs within the tumor of this group. As expected, highest reduction in CSC numbers was found in tumors of the group treated with combination of Orientin and 5FU (Fig. 7g). This led us to check if Orientin in presence of 5FU attenuates CSC-mediated angiogenesis by inhibiting HIF1α in vivo. Like the CSC markers, 5FU also caused the increase in HIF1α and VEGFA expression within the tumor niche. Orientin alone and in combination with 5FU decreased the expressions of the proteins which were overexpressed due to the presence of 5FU. Therefore, it can be concluded that combinatorial treatment has the highest potential to decrease HIF1α and VEGFA expression, which in turn causes inhibition of angiogenesis (Fig. 7h). In a nutshell, all of these data confirmed that Orientin in combination with 5FU significantly inhibited the CSC-mediated angiogenesis in vitro and in vivo.

## Discussion

5FU is considered to be one of the most promising therapeutic agents for CRC. Unfortunately, a number of drawbacks restrict its successful clinical utility. Hepatotoxicity and nephrotoxicity are major hindrances to the successful clinical outcome of 5FU (Ahmad Ansari et al. 2023; Al-Asmari et al. 2016). In this present study, we used Orientin in combination with 5FU to mitigate the drawbacks of 5FU. Orientin is a natural flavonoid with molecular weight 448.38 g/mol. It showed antioxidant activity by scavenging free radicals, reducing oxidative stress through enhancing levels of glutathione and SOD activity (Xiao et al. 2022). Additionally, previous studies reported that it showed anti-inflammatory properties through modulation of TNF-α, IL-1β and COX-2 (Xiao et al. 2017). It also induces apoptosis, inhibits cell proliferation and suppresses tumor growth through modulation of PKCα/ERK/AP-I/STAT3 and NF-κB pathways (Thangaraj and Vaiyapuri 2017; Kim et al. 2018). On the other hand, SwissAdme database (http://www.swissadme.ch/) suggested that Orientin has low gastrointestinal absorption rate which indicate that intravenous administration of this drug will be more effective compared to the oral administration. It is widely distributed in different tissues, undergoes hepatic metabolism via CYP450 enzymes and excreted primarily through urine (Tan et al. 2013; Chagas et al. 2020; Zhang et al. 2007).

Mounting evidences suggest that 5FU induces toxicities mainly in a dose dependent manners (He et al. 2003; Zhang et al. 2018). Our study showed that Orientin treatment enhances the chemosensitization of 5FU to CRC cells when used in combination. Orientin can reduce effective dosage (IC_50_) of 5FU by almost 50% in case of combinatorial treatment compared to the individual treatment (Fig. 1). The present data indicated that Orientin may also inhibit the toxicity developed due to 5FU treatment. Orientin can ameliorate 5FU induced hepatotoxicity as well as nephrotoxicity when administered in combination with 5FU in vivo by normalizing ROS, LPO and phase II detoxifying enzymes in liver and creatinine, BUN in kidney, which suggests that it may reduce the off-target effect of 5FU (Fig. S1).

Another major drawback of 5FU is that it fails to eradicate CSCs and therefore recurrence occurs after few years (Touil et al. 2014). The main reasons for the failure of this conventional chemotherapy in cancer treatment could be explained by the influence of cancer stem cells (CSCs) as it can cause cancer relapse, metastasis, multidrug resistance, and radiation resistance which in turn give rise to new tumors (Samanta et al. 2023). Additionally, recent study has revealed that CSCs have an important role in the development of tumor vasculature (Chen et al. 2020). Angiogenesis, one of the crucial hallmarks of cancer, not only provides adequate amount of nutrition to the malignant cells within the tumor niche but also helps them to migrate into distal organs (Cao et al. 2020). Mounting evidences suggest that overexpressions of stemness-maintaining factors are positively correlated with angiogenesis that promotes aggressiveness in different cancers (Lizárraga-Verdugo et al. 2020; Adini et al. 2013).

Along with these, CSCs secrete angiogenic factors like VEGF and contribute to the vessel recruitment during tumorigenesis. Therefore, CSCs can be considered as the most promising targets for cancer treatment in future. The presence of CSC populations within CRC niche increases the resistance to several treatment strategies. This represents a significant clinical challenge in the fight against CRC. Recent studies have confirmed an association between the presence of CSCs and its chemo-resistance properties (Zhou et al. 2021). Moreover, in advanced CRC, resistance to individual inhibitors of signalling pathways could occur due to enrichment of CSCs. Thus, efforts are underway to find more specific non-toxic molecule which could target signalling pathways to prevent the generation of CSCs and maintenance of its stemness nature.

Several attempts have been conducted to overcome these hurdles by combining CSC targeting agents with conventional chemotherapy. Recently it has been reported that, RO4929097 which target CSCs by inhibiting γ-secretase showed potent anti-tumor activity in preclinical trails but did not show enough potential when used as monotherapy against metastatic CRC patients (Strosberg et al. 1990; Yang et al. 2020). Another CSCs targeting agent, Napabucasin in combination with FOLFIRI and Bevacizumab showed satisfactory effect against CRC (Bendell et al. 2017). In phase III clinical trial several side effects of this treatment were noticed among p-STAT positive patients (Jonker et al. 2018). Another report showed that addition of Bevacizumab to 5FU based chemotherapy did not provide any significant benefit compared to only 5FU based first line chemotherapy (Passardi et al. 2015). Hence, a new therapeutic combination is highly needed to target CSC population in CRC. CD133^+^ CSCs purified from the human CRC specimen can escape 5FU treatment (Todaro et al. 2007). Paschall et al. proved that CD133^+^ and/or CD44^+^ population was increased in 5FU resistant CRC cells (Paschall et al. 2016). In this present study, we also found that after administration of 5FU, percentage of CD44^+^/CD133^+^ cells which is considered as CSC population was increased in CRC (Fig. 2). Another study also proved that 5FU resistant cancer cells show an upregulation of CSC marker proteins like CD44, OCT4 and NANOG (Kulsum et al. 2017). Besides, those resistant cells show significantly increased sphere forming and colony forming ability indicating that the CSC population may escape 5FU treatment (Kulsum et al. 2017). According to our findings, administration of 5FU sustains the CSC maintenance factors like OCT4, SOX2 and NANOG within CSC population which is corroborated with the previous findings (Fig. 2) (Chlebowski et al. 1986). On the other hand, combinatorial treatment of Orientin and 5FU significantly decreased the colorectral CSC population and stem cell maintaining factors which suggests that Orientin has potential to attenuate 5FU induced CSC enrichment (Fig. 2).

**Fig. 8:**
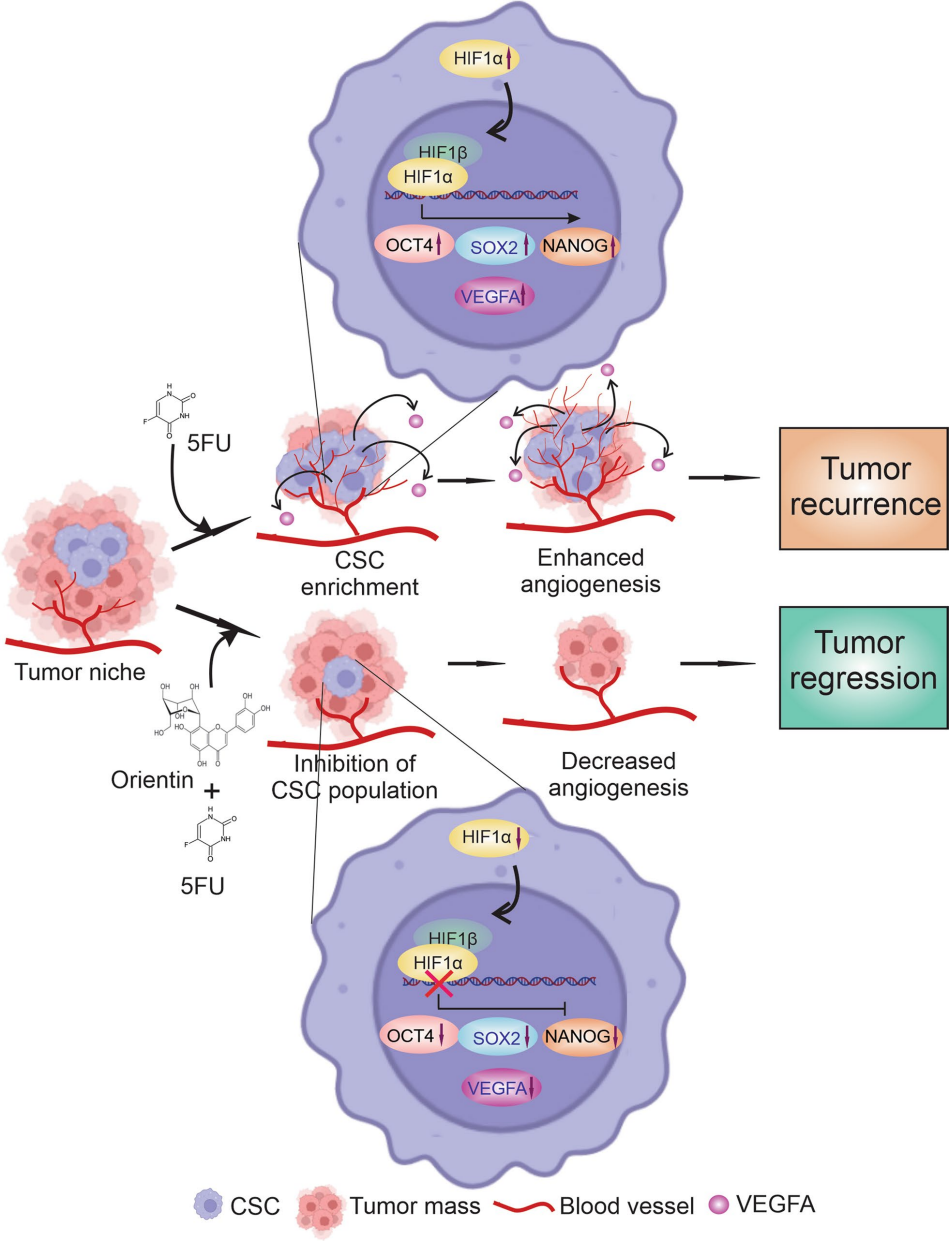
Schematic illustration of CSC mediated angiogenic regulation upon 5FU and combinatorial treatment to colorectal cancer

Along with promoting growth and proliferation of CSCs, another crucial cause to limit the clinical applications of 5FU is enhancement of tumor angiogenesis (Albertsson et al. 2006). It is established that production of ROS, NO and LPO has crucial role in promoting angiogenesis (Morbidelli et al. 2004; Ushio-Fukai and Nakamura 2008; Clemente et al. 2020). Our findings showed that 5FU-induced CSCs promote formation of blood vessels by producing ROS, NO and LPO in CSC niche (Fig. 3), whereas, Orientin in combination with 5FU significantly decreased the formation of blood vessels via normalization of ROS, NO and LPO production (Fig. 3). Therefore, further investigations were focused to elucidate the exact molecular mechanism responsible for the anti-angiogenic effect of Orientin in CSCs. Generation of ROS is associated with upregulation of HIF1α, a key molecule behind angiogenic progression (Cao et al. 2020). HIF1α plays a critical role in stimulating angiogenesis via upregulating VEGF in different cancers (Ghosh et al. 2022). Off note, HIF1α may be associated with the development of 5FU resistance in CRC as patients who are non-responders to 5FU have elevated level of this protein compared to the responders (Fig. S2). Our in silico study showed that Orientin can inhibit activity of HIF1α by binding at the PAS domain of this protein (Fig. 4). This domain is responsible for maintaining the functionality of HIF1α. In vitro findings also confirmed that Orientin alone and in combination with 5FU downregulates expression of 5FU induced HIF1α (Figs. 5–6). Besides, 5FU indulges CSC-mediated VEGFA secretion, which in turn promotes angiogenesis. Another study also revealed that 5FU infusion causes VEGFA mediated angiogenesis which is corroborated with our findings (Albertsson et al. 2009). Our study proved that Orientin in combination with 5FU significantly attenuated CSC-mediated angiogenesis by inhibiting HIF1α mediated VEGFA secretion (Fig. 6). Notably, no significant change in the proliferation of endothelial cells was observed between Orientin and VEGFA neutralizing antibody treatment suggesting that inhibition of angiogenesis by Orientin is comparable to VEGFA neutralizing antibody treatment. Consistent with the in vitro findings, 5FU also stimulated CSC subpopulation as well as tumor angiogenesis in BALB/c mice, which may encourage tumor growth in long-term by providing adequate amount of nutrition to the cells (Fig. 7). However, this drawback of 5FU can be normalized by Orientin treatment in combination with 5FU in vivo (Fig. 7). Taken together, while increasing chemosensitization of 5FU, this combinatorial treatment also significantly attenuates 5FU induced CSC mediated angiogenic progression by inhibiting HIF1α (Fig. 8). According to our in silico study Orientin inhibits activity of HIF1α by binding at its PAS domain. Pas domains are mainly responsible for heterodimerization of this protein as HIF1α binds with HIF1β with the help of this domain (Park et al. 2006). Transcription factor, HIF1α is converted to its active form due to this heterodimerization (Park et al. 2006). Besides inhibition of its activity, a sharp decrease in HIF1α’s expression was noticed after Orientin treatment in vitro. However, the cause behind this alteration in HIF1α expression is not fully known. Various evidences suggest that NF-ĸB can upregulate expression of HIF1α, whereas HIF1α also can upregulate expression of NF-ĸB (Cummins and Taylor 2005; D’Ignazio et al. 2017; Uden et al. 2008). According to our findings Orientin treatment also significantly decreases expression of NF-ĸB in CSCs (Fig. 6). These findings indicated that Orientin inhibits activity of HIF1α, which in turn decreases expression of NF-ĸB. As a consequence, expression of HIF1α gets decreased via this feedback loop existing between NF-ĸB and HIF1α in CSCs which in turn decreases expression of VEGFA resulting in inhibition of CSC-mediated angiogenesis.

## Conclusions

Natural flavonoid Orientin could be a better option for combining with 5FU to achieve the goal of inhibiting CSC-mediated angiogenesis and developing a therapeutic module which is not only as effective as anti-angiogenic antibodies, but also non-toxic and affordable. Orientin makes CRC cells more sensitive to 5FU by reducing its effective dosage and normalizes 5FU induced hepatotoxicity and nephrotoxicity. It also efficiently inhibits 5FU mediated HIF1α expression in combination with 5FU resulting in inhibition of CSC-mediated tumor angiogenesis, which in turn, may provide an effective regimen to restrict cancer progression and relapse. Considering the results presented in this report, we propose a novel combination of 5FU and Orientin to be developed as an efficient therapeutic approach for more effective treatment protocol for CRC by interfering with CSC-mediated angiogenesis and tumorogenesis.

## Supplementary Information

Supplementary Material 1.

Supplementary Material 2.

## Data Availability

No datasets were generated or analysed during the current study.

## Abbreviations

CRC: Colorectal cancer
CSC: Cancer stem cell
Ori: Orientin
5FU: 5-Fluorouracil
HIF1α: Hypoxia Inducible Factor 1α
VEGFA: Vascular Endothelial Growth Factor A
HUVEC: Human Umbilical Vein Endothelial Cells
ROS: Reactive oxygen species
CES: CSC enriched spheres
CM: Conditioned medium
AFM: Atomic force microscopy
MACS: Magnetic-activated cell sorting
CAM: Chorioallantoic membrane
DCFH-DA: 2′,7′-Dichlorofluorescein diacetate
NO: Nitric oxide
LPO: Lipid Peroxidation
HPA: Human Protein Atlas
ROC: Receiver Operating Characteristic Curve
RCSB: Research Collaboratory for Structural Bioinformatics
PDB: Protein Data Bank
RMSD: Root mean square deviation
RMSF: Root mean square fluctuation
ALT: Alanine aminotransferase
AST: Aspartate aminotransferase
ALP: Alkaline phosphatase
BUN: Blood Urea Nitrogen
GST: Glutathione-S-transferase
SOD: Superoxide dismutase
CAT: Catalase
GPx: Glutathione peroxidase
GSH: Glutathione
NADPH: Nicotinamide adenine dinucleotide phosphate hydrogen
DTNB: 5,5′-Dithiobis-2-nitrobenzoic acid
TNB: 5,5′-Thiobis-2-nitrobenzoic acid
MST: Mean Survival Time
