# Supplementary material for: Natural flavonoid Orientin restricts 5-Fluorouracil induced cancer stem cells mediated angiogenesis by regulating HIF1α and VEGFA in colorectal cancer
Source: Mol Med. 2025 Mar 5;31:85. doi: 10.1186/s10020-024-01032-1 (PMC11881437; doi:10.1186/s10020-024-01032-1)

**Supplementary Figure Legends:**

**Fig. S1. Quantitation of CSC population in HCT116 cell line and HCT116 cells derived CES.** Expression profile of both CD44 positive (X axis) and CD133 positive (Y axis) in HCT116 cell line and HCT116 cells derived CES are characterized by flow cytometry. Cell populations are identified based on PE-conjugated anti-CD133 and FITC-conjugated anti-CD44 antibodies where percentages of double positive cell population are represented in the upper right quadrate of each plot.

**Fig. S2. Determination of expression of HIF1α and VEGFA in human tissues** a) Protein expression data from HPA shows a heterogeneous expression of HIF1α and VEGFA in different human tumor tissues. b) Box plot represent HIF1α and VEGFA gene expression in colorectal cancer comparing normal and tumor tissues c) The differential expression of HIF1α (left panel) and VEGFA (right panel) are evaluated by ROC analysis in a validation set of 379 responders and 279 non-responders colorectal cancer patients who received 5FU.

**Fig. S3:** **Determination of toxicity of Orientin *in vivo*.** a) various dosages of orientin treatment decreased i) LPO level and increased ii) GST, iii) GPx, iv) GSH, v) SOD and vi) CAT activity in hepatic tissue of mice after 28 days of treatment. Data are represented as mean ± SD, (n = 6), * indicates p < 0.0001 significantly different from vehicle control (VC) group (one-way ANOVA followed by Tukeys Multiple Comparison Test), b.w = body weight. b) Images represent histopathology of i) liver, ii) heart, iii) lung and iv) kidney of vehicle control (VC) and 5 mg/kg b.w Orientin (Ori) treated mice done by H&E staining. All the images are captured under bright field microscope with 20X magnification.

**Fig. S4. Attenuation of 5FU induced hepatotoxicity by combinatorial treatment in mice.** Alteration in expression of phase II detoxifying enzymes i) GSH, ii) GST, iii) GPx, iv) SOD and v) CAT activity and vi) LPO, vii) ROS and viii) NO level in hepatic tissue of mice after 10 days treatment of Orientin, 5FU, Orientin and 5FU in combination. Data are represented as mean ± SD, n = 6. ^#^ indicates p< 0.0001 significantly different from vehicle control (VC) group; * indicates p< 0.0001 significantly different from only 5FU-treated group (one-way ANOVA followed by Tukeys Multiple Comparison Test).

**Fig. S5. Mitigation of 5FU induced hepatotoxicity and nephrotoxicity by histopathological analysis.** Images represent histopathology of a) liver and b) kidney of vehicle control (VC), Orientin (Ori), 5FU and combination of Orientin and 5FU (Ori +5FU) treated mice done by H&E staining. All the images are captured under bright field microscope with 20X magnification.

**Supplementary figures:**

**Fig. S1.**

**
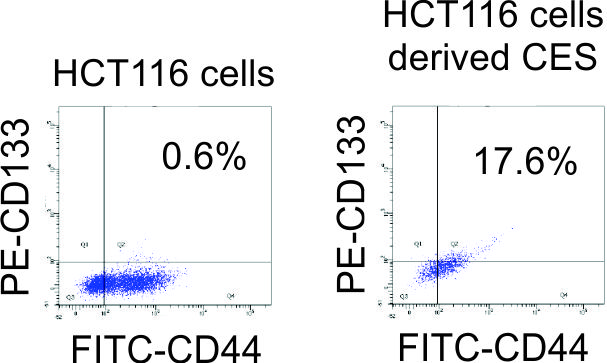
**

**Fig. S2.**

**
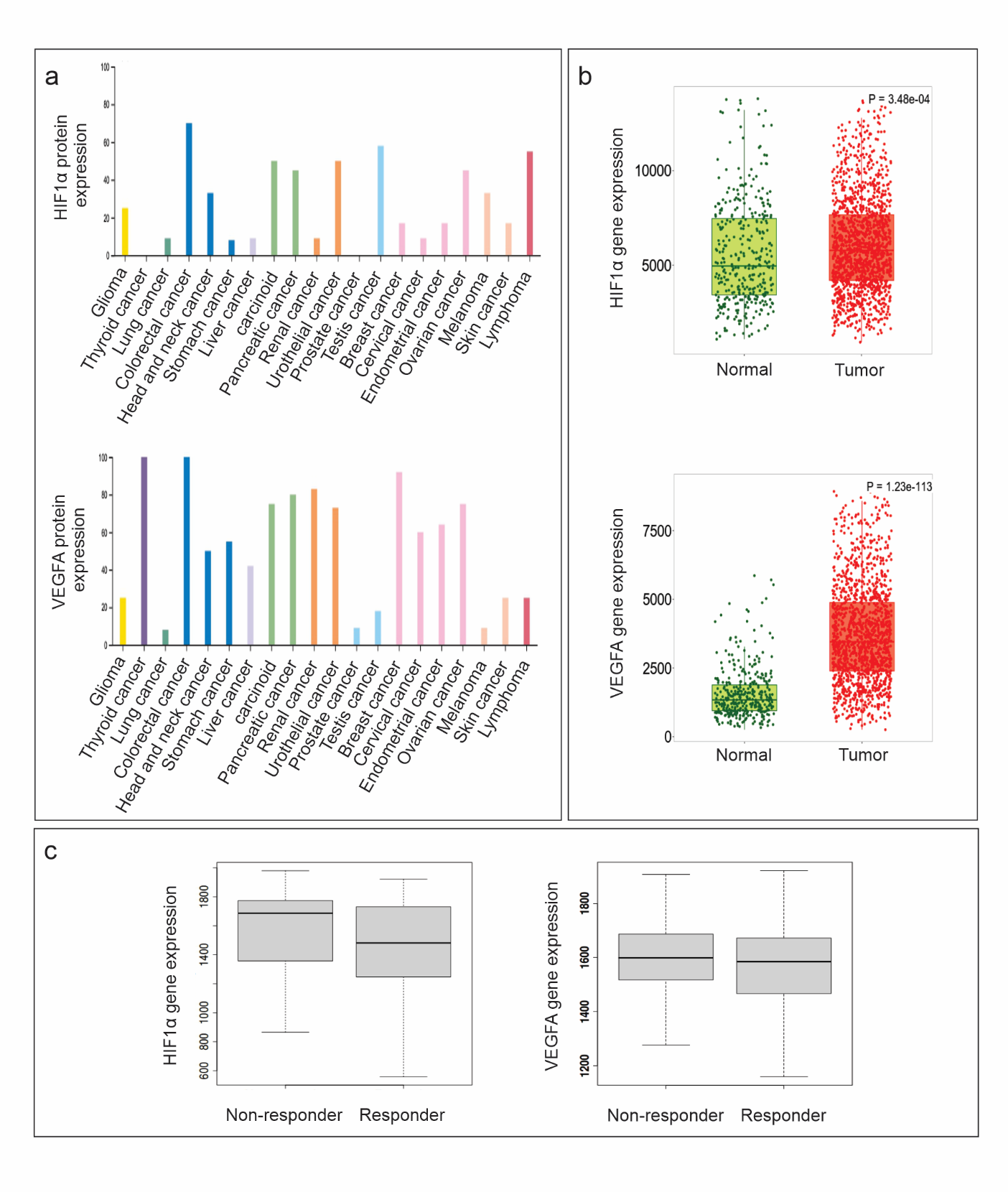
**

**Fig. S3.**


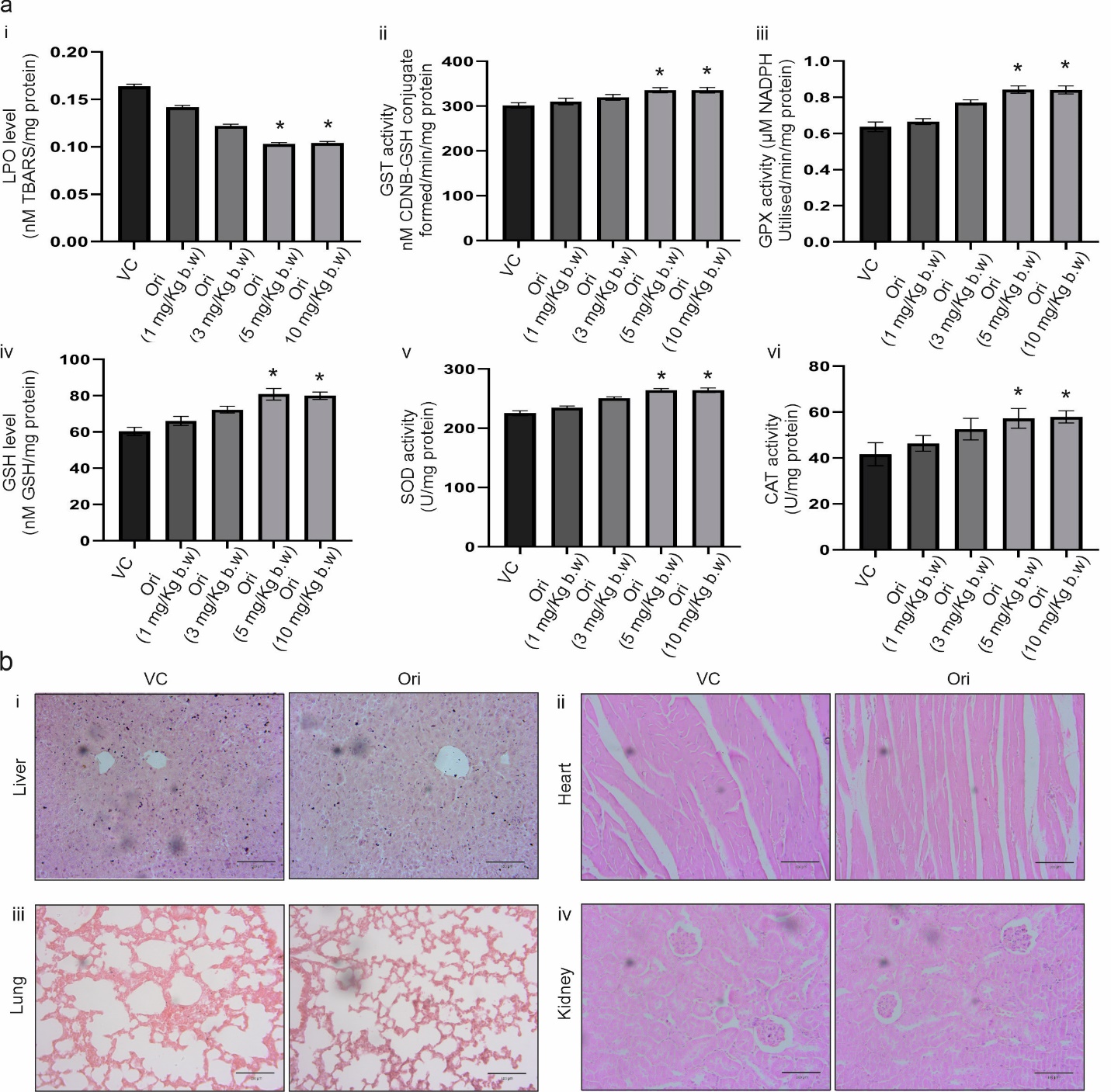


**Fig. S4.**

**
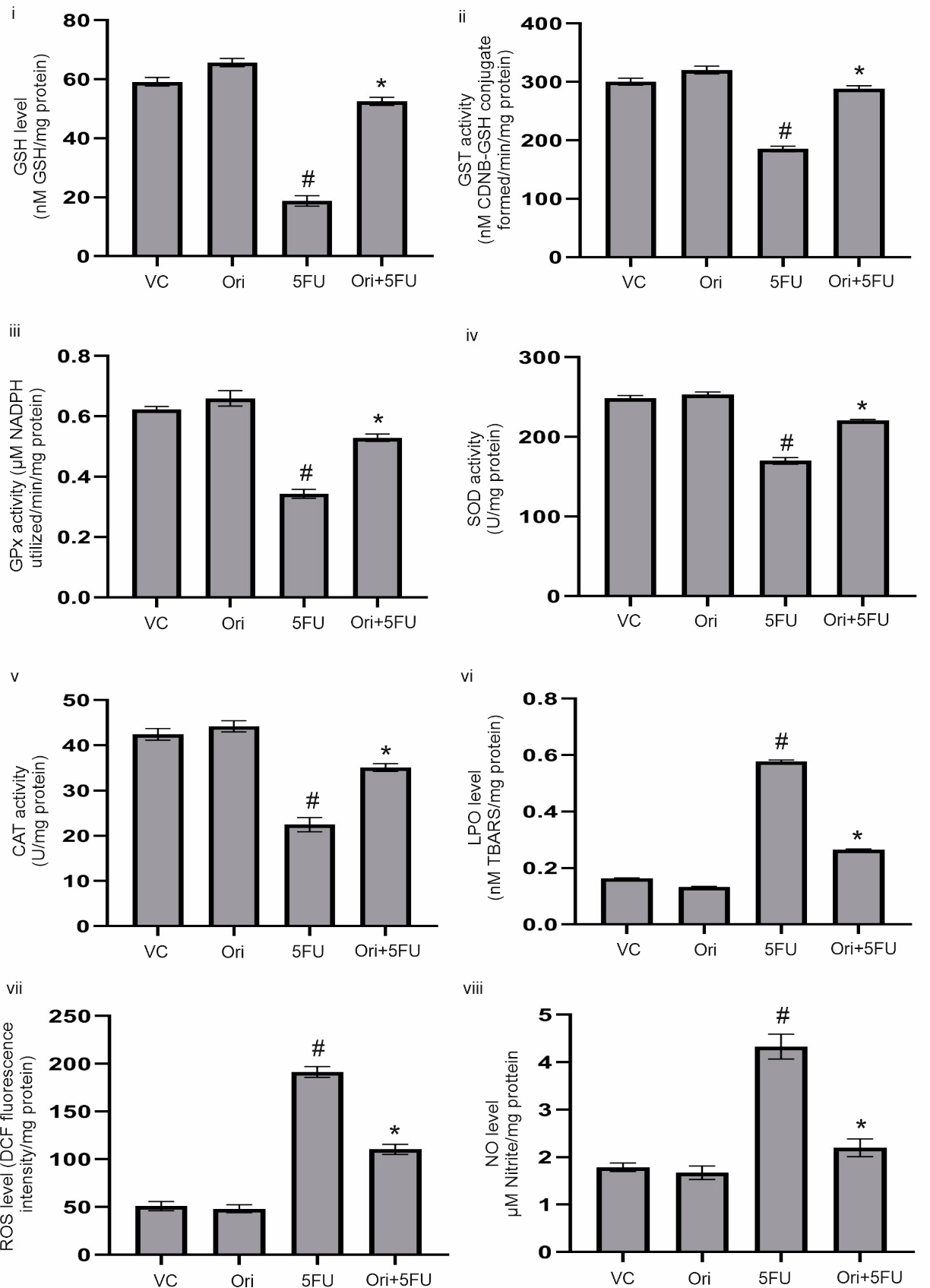
**

**Fig. S5.**


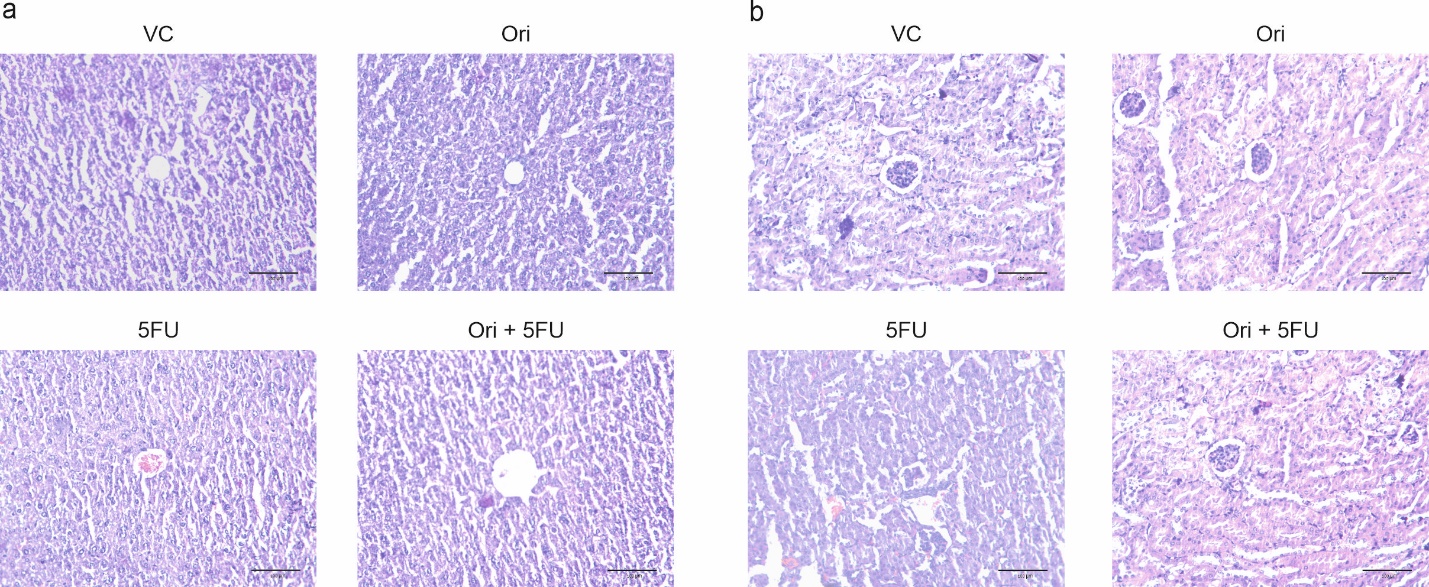

Supplement: Supplementary file 1 — Supplementary Material 1. [file 10020_2024_1032_MOESM1_ESM.docx]
